# Systems biology and functional assessments of human iPSC-cardiomyocyte models of insulin resistance captures key hallmarks of diabetic cardiomyopathy

**DOI:** 10.2337/db25-0204

**Published:** 2025-11-01

**Authors:** Ryan D. Carter, Ujang Purnama, Marcos Castro-Guarda, Claudia N. Montes-Aparicio, Anandhakumar Chandran, Richard Mbasu, Maxwell Ruby, Charlotte Daly, Kirsti Brisk, Helen C. Christian, Jack J.J.J. Miller, Francesca M. Buffa, Lisa C. Heather, Carolyn A. Carr

**Affiliations:** 1 Department of Physiology, Anatomy and Genetics, University of Oxford, Parks Road, Oxford, UK; 2 Novo Nordisk Research Centre Oxford, Innovation Building, University of Oxford, Old Road Campus, Oxford, UK; 3 Department of Clinical Medicine, Aarhus University, Denmark; 4 Department of Oncology, Medical Sciences Division, University of Oxford, Old Road Campus Research Building, Roosevelt Drive, Oxford, UK; 5 Department of Computing Sciences, Bocconi University, Bocconi Institute for Data Science and Analytics (BIDSA), Milano, Italy

## Abstract

Human-centric models of diabetic cardiomyopathy (DbCM) are needed to provide mechanistic insights and translationally-relevant therapeutic targets for patients with diabetes. A systems biology approach using insulin resistant (IR) 2D human induced pluripotent stem cell-derived cardiomyocytes (hiPSC-CMs) and 3D engineered heart tissue (EHT) provides a comprehensive evaluation of dysregulated pathways and determines suitability as a translationally-relevant model of DbCM.

Culturing hiPSC-CMs in 2D or 3D EHT in “IR” media induced insulin resistance and activated multiple pathways implicated in DbCM, including metabolic remodelling, mitochondrial dysfunction, extracellular matrix remodelling, endoplasmic reticulum stress and blunted response to hypoxia, assessed using transcriptomics and proteomics. Metabolic flux measurements in both IR 2D and 3D platforms demonstrated increased fatty acid oxidation and lipid storage, whereas glucose metabolism was downregulated. Modelling DbCM in 3D EHTs conferred additional metabolic and functional advantages over the 2D hiPSC-CM, demonstrating impaired contractility and muscle architecture. Metformin treatment improved both contractility and metabolic function, demonstrating the utility of IR EHT for drug assessment.

In conclusion, IR 2D and 3D hiPSC-CM models effectively capture key DbCM features. However, 3D EHT provide additional insights into physiological and structural modifications. This highlights the potential of IR EHT for both mechanistic studies and drug screening in DbCM.

**Figure:**
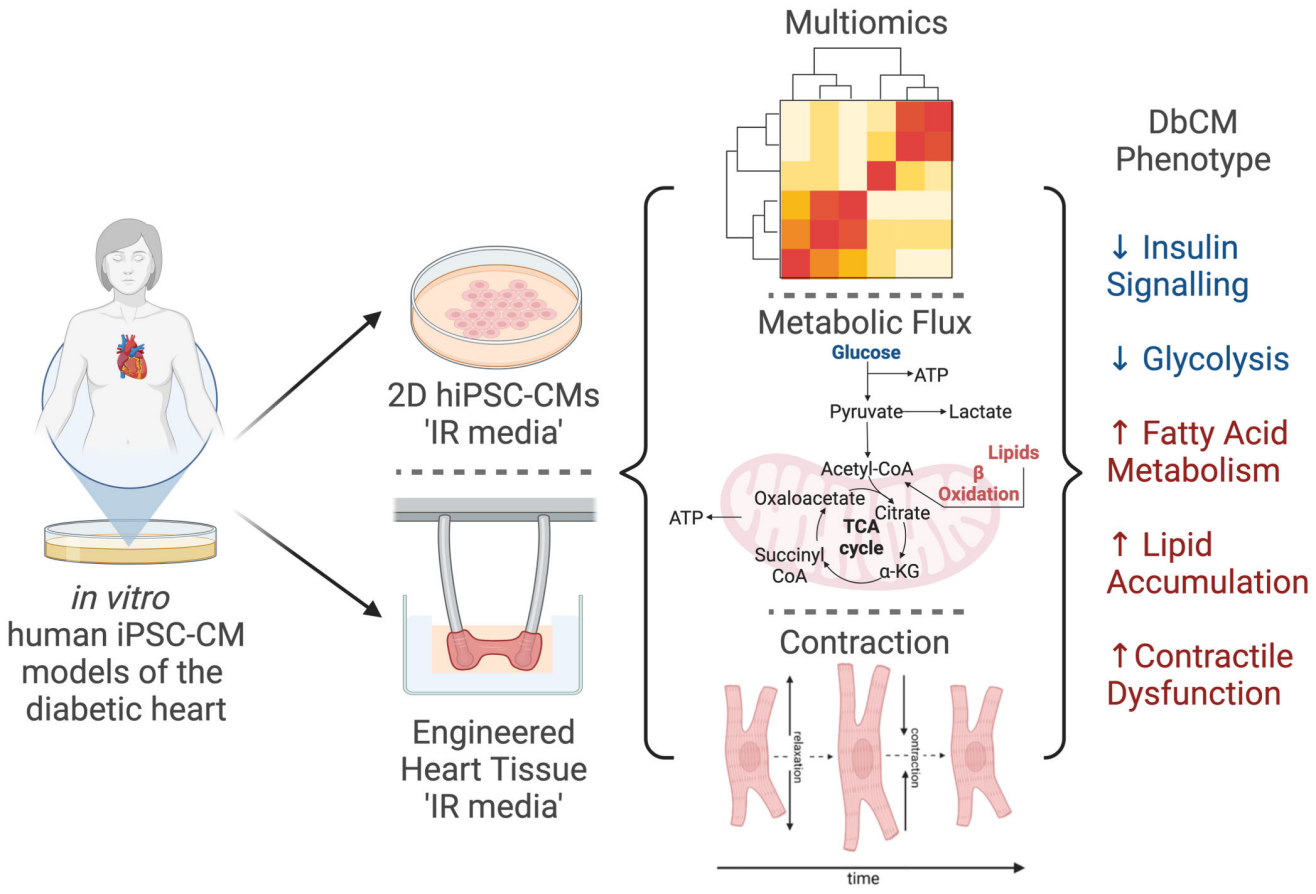

## Introduction

Type 2 diabetes mellitus (T2DM) disrupts systemic metabolic homeostasis, increasing cardiovascular disease (CVD) risk and cardiovascular mortality^1^. The interplay between T2DM and CVD involves shared risk factors including hypertension and coronary artery disease, alongside direct effects on cardiomyocyte structure and function, termed diabetic cardiomyopathy (DbCM)^2^. DbCM is characterised by diastolic dysfunction and left ventricular hypertrophy, arising from structural pathologies including fibrosis and altered myofilament function^3^. These changes are driven by profound alterations in cardiomyocyte metabolism, including insulin resistance (IR), toxic lipid accumulation, mitochondrial dysfunction, elevated fatty acid (FA) metabolism, and endoplasmic reticulum stress^4^.

Although DbCM is widespread^5^, the development of therapies is limited by an unmet need for preclinical models that reflect human pathophysiology^6,7^. Significant inter-species differences in cardiac metabolism, ion channel function and inflammatory responses mean that findings from rodent models often fail to translate to patients, leading to costly and unsuccessful clinical trials^8,9^. Complementary human-centric models, such as human induced pluripotent stem cell-derived cardiomyocytes (hiPSC-CMs), offer a viable alternative^10^. HiPSC-CMs can be grown in 2D, or 3D as organoids or engineered heart tissue (EHTs)^11,12^, with the latter 3D models showing greater morphological, metabolic, and sarcomeric maturation of cardiomyocytes^13^, traded-off against greater time- and cost-intensive generation. Although hiPSC-CMs have been used extensively in modelling monogenic diseases^14^, using them to study complex multifactorial, lifestyle-related diseases like T2DM has been limited^6^.

Initial attempts to model the diabetogenic environment *in vitro* have used short-term exposure to high glucose or FA^15-19^. However, a robust model requires a multifactorial approach that captures the chronic, concurrent impact of hyperglycaemia, hyperlipidaemia, and hyperinsulinemia. Our approach first enhances the maturation of hiPSC-CMs by culturing them with low FA concentrations, thereby providing a more physiologically relevant baseline for disease studies^13^. We apply our multifactorial, longer-term IR protocol to these matured cells in both 2D cultures and advanced 3D EHTs, to assess the ability to recapitulate the hallmarks of DbCM. This was assessed using multi-omics analyses to gain a systems-level understanding of the pathways regulated in our hiPSC-CMs in response to IR. This broader perspective, which extends beyond single-pathway evaluations, provides a more comprehensive view of how different aspects of the diabetic milieu converge to affect cardiac function in DbCM.

Our findings show that 2D and 3D IR models can replicate key hallmarks of DbCM. Additionally, in 3D EHT, physiological parameters such as contractility were also significantly impacted by IR. Importantly, we demonstrate that treatment with metformin reverses many of these metabolic and contractile abnormalities, highlighting the potential of IR EHTs as platforms for mechanistic studies and translationally-relevant drug screening for DbCM.

## Methods

### hiPSC-CM differentiation

**Figure 1:**
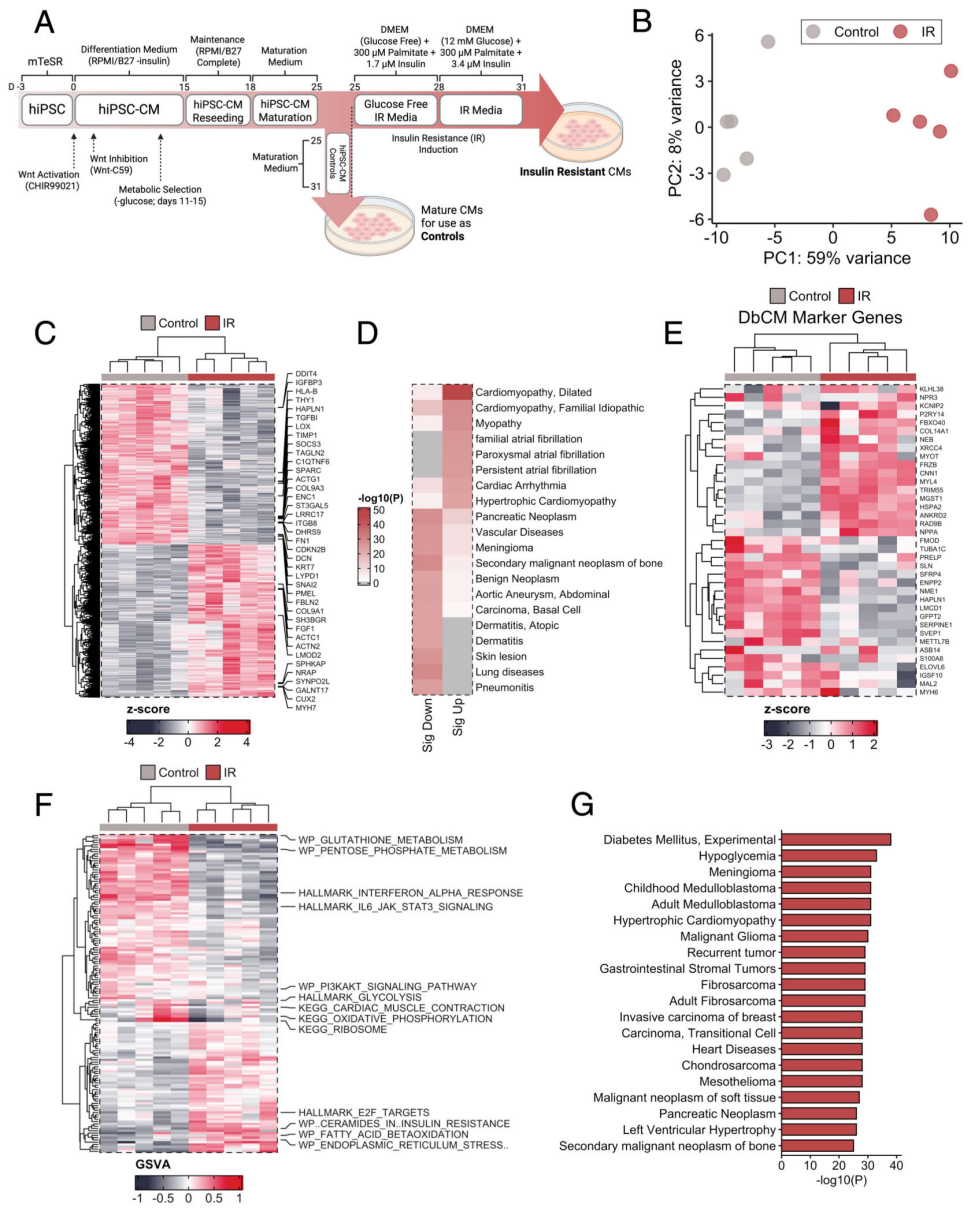
Culturing 2D hiPSC-CM in an IR media captures many characteristics of Diabetic Cardiomyopathy. (A) Schematic of the protocol to generate insulin-resistant (IR) and control hiPSC-CMs, involving a 15-day differentiation, a 7-day maturation, and a 6-day insulin resistance induction period. (B) Principal component analysis (PCA) of transcriptomic data demonstrates a clear separation between IR and control groups along the first principal component (*n* = 5 samples per condition). (C) Heatmap showing significantly altered genes in IR hiPSC-CMs, as identified by differential expression analysis (DESeq2, FDR < 0.05). (D) Disease pathway enrichment analysis highlights cardiomyopathy as a significantly overrepresented pathway among the differentially expressed genes. (E) The expression of DbCM patient marker genes^40^ stratified control and IR IPSC-CMs. (F) Single-sample summary scores for non-overlapping, enriched pathways, ranked by differential expression, distinguish the IR and control groups, with DbCM-related terms being prominent. (G~) Leading-edge genes from selected pathways (detailed in the main text) show the most significant overrepresentation for diabetes and DbCM-related disease terms.

IMR90 (ECACC Catalogue No. 85020204 female Caucasian foetal lung origin) human induced pluripotent stem cells (hiPSCs) were differentiated into hiPSC-CM over 15 days by modulating Wnt signalling^20^ as previously described^21^ (Figure 1A). Confluent hiPSCs cultured in TeSR-E8 media on Reduced Growth Factor Matrigel-coated plates were moved to RB- media (RPMI with 1% B27 -insulin) containing CHIR99021 (6 μM) for two days. The media was then replaced with RB- only for one day, followed by a two-day treatment with RB- containing Wnt-C59 (2.5 μM). From day five, cells were maintained in RB- media. For metabolic selection of CMs, glucose-free RB- media was used on days eleven and thirteen. Finally, on day fifteen, the cells were switched to RB+ media (RPMI with 1% B27 complete). This differentiation protocol resulted in >80% cardiomyocytes^13^. The cells were differentiated in six-well plates, and cells from different wells in the plate were used as technical replicates. For each experiment, the ‘n’ number refers to separate differentiations as biological repeats.

### Generation of 3D Engineered Heart Tissue

EHTs were generated using the protocol as previously described ^22^ (Figure 5A). HiPSC-CMs were differentiated and dissociated on DD15 into single cells. For a single EHT, 10^6^ hiPSC-CMs were re-suspended in 94.56 μL of hydration media (DMEM incl. 1% penicillin–streptomycin, 10% FBS, 1% glutamine), 6.68 μL 2 x DMEM (20% 10 x DMEM (1.3 g DMEM powder in 10 mL ddiH_2_O), 20% horse serum, 0.12 μL Y-27632 rock inhibitor, 9 mM), 12 μL reduced growth factor Matrigel,3.04 μL of a mixture of fibrinogen (200 mg/ml in 0.9% NaCl) and aprotinin (S33 mg/ml added to a concentration of 0.1 mg/ml). To this master mix, 3.6 μL bovine thrombin (Biopur, 100 U/mL) was added immediately before pipetting the solution into a pre-cast 2% agarose mould prepared using Teflon spacers where it solidified around two silicone posts over 90 minutes. An additional 500 μL of hydration media was added for 30 minutes.

### 2D and 3D hiPSC-CM maturation protocol

Once beating, the 2D hiPSC-CMs and 3D EHTs were cultured in maturation media for seven days, based on our previous publications^13,21^. The maturation media comprises DMEM (1 g/L glucose) supplemented with 10% horse serum, 5 μg/mL vitamin B12, 0.5 mM ascorbic acid, 0.84 uM biotin, 1.7 μM insulin, 80 μM oleic acid: BSA (2:1), 1x MEM non-essential amino acids, and 0.1× penicillin-streptomycin.

### Inducing Insulin Resistance in hiPSC-CM and hypoxic exposure

IR was induced over six days using a two-step media protocol^21^. IR media comprised DMEM supplemented with 1X nonessential amino acids, 0.5 mM ascorbic acid, 5 μg/mL vitamin B12, 0.84 μM biotin, 0.1× penicillin/streptomycin, and 10% horse serum. Mature hiPSC-CMs or EHTs were cultured for three days in glucose-free IR media containing 0.3 mM palmitic acid (bound to BSA) and 1.7 μM insulin, and then switched to glucose-enriched IR media containing 12 mM glucose, 0.3 mM palmitic acid (bound to BSA) and 3.4 μM insulin for a further three days. Control samples were maintained in maturation media for 6 days. A subset of control and IR EHTs was treated with 0.5 mM metformin for the final 24 hours.

In a separate experiment, mature hiPSC-CMs were cultured in DMEM (1g/L glucose and the same vitamins as above) with or without palmitate (0.4 mM bound to BSA) for 24 hours. For hypoxia experiments, hiPSC-CMs were incubated for 16 hours in 2% O_2_, 5% CO_2_ and 37°C, whereas normoxic controls were in 21% O_2_, 5% CO_2_ and 37°C. Hypoxic cells were rapidly lysed after removal from the incubator to minimise reoxygenation^21^.

### RNA isolation and quantitative PCR

RNA was extracted from cells using the Qiagen RNeasy Mini kit with QIAzol lysis agent. For quantitative PCR (qPCR), cDNA was synthesised using a high-capacity RNA-to-cDNA kit before analysis using the Step-One Plus Real-Time PCR system with Power SYBR Green PCR Master Mix. Relative gene expression was calculated using the 2−ΔΔCT method and normalised to the housekeeping gene Ubiquitin C (Table S1).

### Transcriptomics mapping, quantification, and differential expression analysis

mRNA from 2D hiPSC-CMs and EHTs was sequenced by Illumina: for hiPSC-CMs, Novogene performed stranded, paired-end (150 bp) poly-A enrichment sequencing; for EHTs, Illumina 3’ QuantSeq was conducted by Novo Nordisk Research Centre Oxford (NNRCO). Post-sequencing, FastQC was used for quality control, and cutadapt removed adapters and low-quality reads^23^. hiPSC-CM transcripts were quantified with Salmon^24^ against a gencode v44 index, while EHT reads were mapped with STAR^25^ using Ensembl v109.

Differential expression analysis was performed with DESeq2, removing genes with low counts. Counts were normalised using variance stabilisation transformation (VST) for exploratory analysis, including principal component analysis (PCA). Log-fold changes were refined using apeglm Bayesian shrinkage^26^. Statistical significance was defined as a Benjamini-Hochberg adjusted p-value < 0.05. For the EHT data, surrogate variable analysis^27^ identified a latent batch effect, which was incorporated into the DESeq2 design or corrected using the Limma package^28^. Z-scores from normalised expression were used to visualise pathway expression in heatmaps. Hierarchical clustering of both pathways (rows) and samples (columns) was performed using Euclidean distance with the complete linkage method. Differential transcript usage (DTU) was assessed using the DTUrtle R package^29^ to identify proportional changes in gene transcript composition. DTUrtle implements the DRIMseq methodology, incorporating false discovery rate (FDR) correction via the stageR package and post-hoc filtering to remove transcripts with low expression variance^30^.

### Proteomics quantification and differential abundance analysis

Proteomics analysis was conducted by NNRCO via LC-MS/MS. Protein samples (~100 μg) were prepared in 50 mM ammonium bicarbonate with 10 pmol horse myoglobin and 0.2% RapiGest, reduced with 10 mM dithiothreitol, and alkylated with 15 mM iodoacetamide. Samples were then digested overnight with trypsin (1:50 ratio), quenched with 0.5% trifluoroacetic acid for 45 minutes, and reconstituted in 0.1% formic acid before loading 500 ng for LC-MS/MS analysis.

Peptides were identified using PEAKS DB^31^, with protein abundance calculated from the summed AUC of extracted ion chromatograms. Subsequent analysis used the R packages DEP^32^ and proDA^33^. DEP was used for filtering, VST normalisation, and imputation for PCA. Differential abundance analysis was performed with proDA.

### Pathway-based analysis

GSEA was performed with the FGSEA package^34^, ranking genes by differential expression test statistics without filtering low-expression genes to preserve accurate NULL distributions. For proteomics data, UniProt IDs were mapped to gene symbols. Pathways from Wikipathways, KEGG, and hallmark pathways (Molecular Signatures Database^35^) were merged, and FGSEA's collapsePathways function reduced pathway redundancy. P-values were adjusted using the Benjamini-Hochberg method, with FDR < 0.05 as the threshold. Single-sample enrichment analysis via the GSVA^36^ algorithm visualised significant pathways. Over-representation and protein-protein interaction (PPI) analyses were done with Metascape^37^. Tissue-specific gene enrichment was analysed with the TissueEnrich web portal^38^ using the set of non-lowly expressed genes from 2D control and IR samples separately.

### Measurement of substrate metabolism

Glycolytic rates were measured by culturing 2D cells or 3D EHT in DMEM (5mM glucose) supplemented with ^3^H-glucose (0.2 μCi/mL of D-[5-^3^H(N)] glucose) with or without 50 nM insulin for 6 hours, with the ^3^H-tracer released at the enolase reaction. In separate experiments, FA oxidation rates were measured by culturing cells or EHT in DMEM (0.3 mM oleate) supplemented with ^3^H-oleate (0.2 μCi/mL of [9,10-^3^H(N)]-oleic acid) for 8 hours, with the tracer released at complex IV of the ETC. Metabolic flux rates were measured by the rate of conversion of the ^3^H-substrate into ^3^H_2_O, as previously described^13^. Oil red O staining was carried out on EHTs using an Oil Red O staining kit after fixing in 4% PFA (nuclei were stained with DAPI). Glucose and lactate concentrations in cell media were measured using an ABX Pentra 400, before and after 24 hours of culture.

### Measurement of contractile function

Videos of contracting EHTs or 2D iPSC-CM monolayers were recorded for 15–30 s at 60 × magnification with a Canon EOS-100D at room temperature. Recordings were converted to 50 fps TIFF stacks with ffmpeg and analysed in MUSCLEMOTION^39^ version 1.195-197 (FIJI/ImageJ plug-in) to calculate peak-to-peak time (cycle length), contraction duration, relaxation time, time-to-peak and contraction amplitude (Figure S5). All preparations beat spontaneously, so no electrical pacing was applied.

Because each rate metric depends on cycle length, statistical tests were run on the natural-log scale: ln(metric) was modelled as a linear function of ln(peak-to-peak). For single-factor comparisons a log ANCOVA was fitted with group as the fixed factor and ln(cycle length) as the covariate; observations were back-transformed to a common reference cycle length (the geometric mean of the dataset, typically 231–268 ms) for plotting. For two-factor comparisons, mixed-effects models were used: ln(metric) ~ Tissue × Sample + ln(peak-to-peak) + (1 + ln(peak-to-peak) | Tissue: Sample). This allows each tissue–sample combination to have its own cycle-length slope while still borrowing information across groups. Fixed-effect contrasts from these models provide the main culture effect (Tissue), the main treatment effect (Treatment) and their interaction, which give the p-values reported in the legends.

### Western blotting

Cells or EHT were washed with DPBS and cultured for 30 minutes with or without 10 μg/ml of insulin, followed by lysing and snap freezing in lysis buffer. Western blotting antibody details are provided in Table S2.

### Electron Microscopy

EHTs were removed from posts and immersion-fixed in 2% paraformaldehyde/2.5% glutaraldehyde for 2 hours, then stored in 10 % fixative solution. Samples were washed in 0.1M phosphate buffer, treated with 1% osmium tetroxide and stained with 2% uranyl acetate. They were dehydrated in increasing concentrations of ethanol (70-100%) and acetone, and embedded in Spurr’s resin. 50-80 nm sections were cut using a microtome and mounted on 200 mesh nickel grids. Samples were imaged using a JEM1010 JEOL transmission electron microscope with a GANTAN Orius camera. Images were analysed using ImageJ by drawing a region around muscle fibres, identified by filaments organised into sarcomeres.

### Statistical analysis and plots

All statistical analysis was conducted in R (v 4.3.1). The assumption of data normality was first evaluated using the Shapiro-Wilk test. For two-group comparisons, Student's t-tests were used for normally distributed data, while the non-parametric Wilcoxon rank-sum test was employed for data that were not normally distributed. Similarly, for factorial analyses, a two-way ANOVA with Tukey’s post-hoc test was performed on normal data, whereas the Aligned Rank Transform (ART) ANOVA was utilised for non-normal data. Statistical comparisons for gene expression are based on DESeq2 results. Barplots depict the mean and standard deviation, while boxplots show the median and interquartile ranges. Significance levels are denoted as follows: * p < 0.05, ** p < 0.01, *** p < 0.001, and **** p < 0.0001. Illustrations were created with BioRender.com (https://Biorender.com/s5eeg3land/5he10j5).

## Results

### Culturing 2D hiPSC-CM in an “insulin-resistance” media captures many characteristics of DbCM

During the progression of T2DM, the heart is exposed to hyperglycaemia, hyperlipidaemia and hyperinsulinaemia^4^. Therefore, we utilised hiPSC-CM exposed to an 'insulin resistance' (IR) media (Figure 1A) to identify human relevant mechanisms related to DbCM *in vitro*. All cardiomyocytes were first matured with a low concentration of oleate, utilising a protocol developed in-house to activate key oxidative metabolic pathways^13^ (without causing lipid overload (Figure S1)). 2D hiPSC-CMs were cultured for 6 days in control or IR media before RNA sequencing, to undertake a high-dimensional assessment of our model. Our hiPSC-CMs comprise >80% α-actinin expressing cells^13^, and expressed genes were strongly enriched for ‘Heart Muscle’ signatures (Figure S2). PCA revealed a clear separation between groups, with PC1 capturing 59% of the variance (Figure 1B). The IR treatment prompted a robust transcriptomic response, with 4,142 differentially expressed genes (DEGs) identified (Figure 1C) constituting ~23% of all expressed genes (n=18,854), highlighting a substantial transcriptional reprogramming.

Pathway analysis indicated that DEGs were significantly enriched for cardiovascular disease terms, including cardiomyopathies (Figure 1D), and known DbCM patient biomarkers robustly clustered the treatment groups (Figure 1E)^40^. Gene Set Enrichment Analysis (GSEA) of the entire ranked gene list revealed 147 significantly altered pathways, which distinctly separated control and IR groups upon single-sample enrichment and hierarchical clustering (Figure 1F). Notably, the leading-edge genes driving the enrichment of core metabolic and signalling pathways—including glycolysis, oxidative phosphorylation, β-oxidation, ER stress, and PI3K-Akt/insulin signalling—were themselves collectively over-represented in the ‘Experimental Diabetes Mellitus’ gene set (Figure 1G). Our IR protocol did not induce substantial cell death or apoptosis (Figure S1). Taken together, these data demonstrate that our *in vitro* model recapitulates several key transcriptional hallmarks of DbCM.

### The 2D hiPSC-CM model captures the IR phenotype with decreased glycolysis

**Figure 2:**
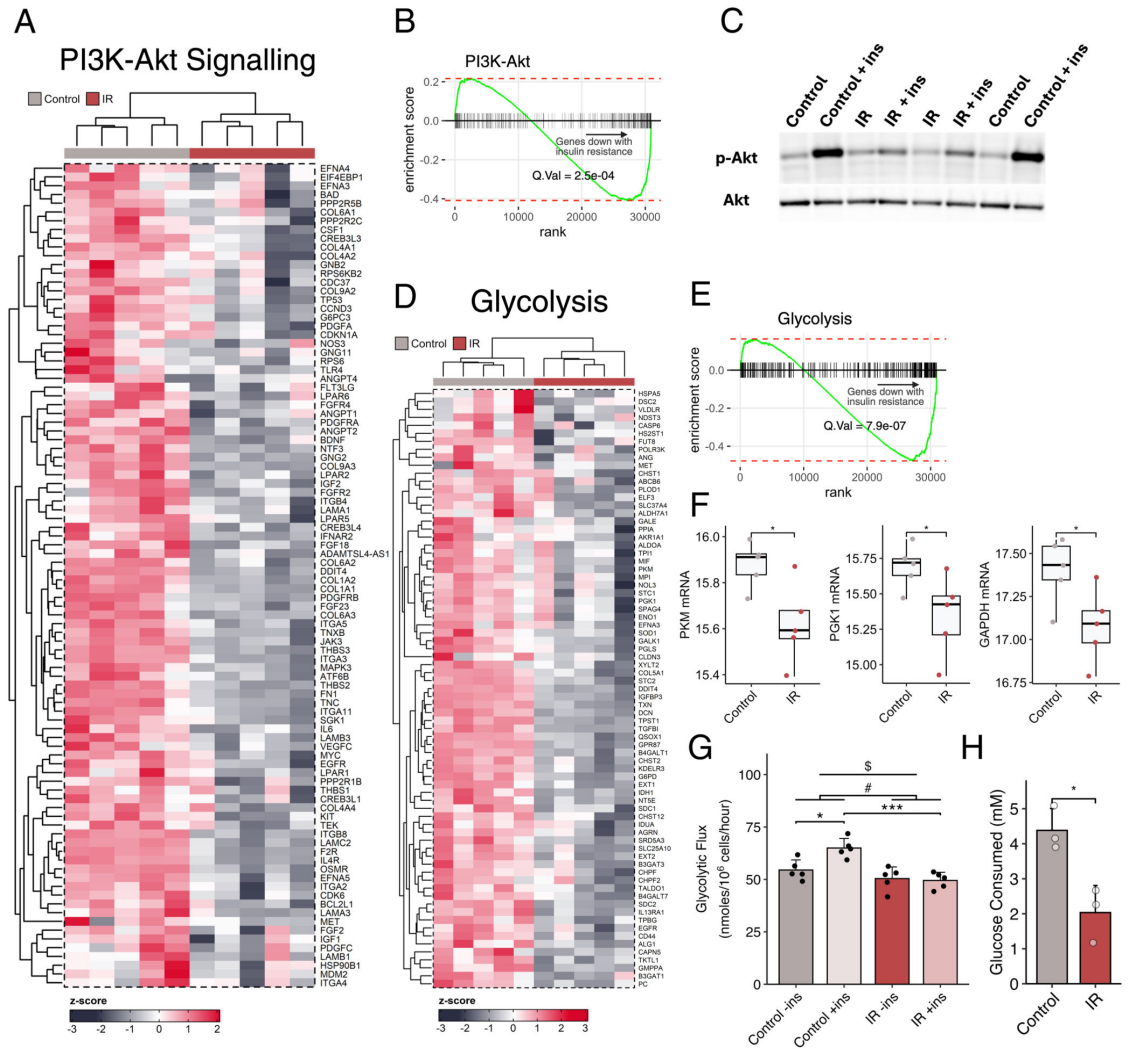
The 2D hiPSC-CM IR model shows blunted insulin signalling and decreased glycolysis. (A, B) Heatmap showing visibly reduced expression of leading-edge genes for the significantly negatively enriched PI3K-Akt pathway (MsigDB: WikiPaths) in insulin-resistant (IR) samples compared with controls. (C) Representative western blot images of phosphorylated Akt (pAkt) and total Akt in control and IR hiPSC-CMs. (D, E) mRNA expression of glycolysis pathway genes (MsigDB: Hallmark) was significantly downregulated in IR hiPSC-CMs, visualised at the pathway level. (F) Boxplots showing significantly reduced mRNA expression of the rate-limiting glycolytic enzymes pyruvate kinase M (PKM), phosphoglycerate kinase 1 (PGK1), and glyceraldehyde-3-phosphate dehydrogenase (GAPDH) in IR cells (median ± IQR; DESeq2, *p < 0.05, *n* = 5). (G) Bar plot of radioisotope flux measurements with and without insulin-stimulation (ins) confirming significantly lower glycolytic rates in IR hiPSC-CMs compared with controls (mean ± SD; two-way ANOVA with Tukey’s post hoc test, *p < 0.05, ***p < 0.001; #*p* < 0.05 main effect of IR; $*p* < 0.05 interaction term; *n* = 5). (H) Bar plot of glucose metabolism by control and IR cells over 24 hours, measured by enzymatic assay (mean ± SD; Student’s t-test sum test, *p < 0.05, *n* = 3).

Our transcriptomic data pointed to defective insulin signalling, a core feature of T2DM. Accordingly, the PI3K-Akt pathway was significantly downregulated in our IR model, with the expression of its leading-edge genes robustly separating the treatment groups (Figure 2A, B). To validate insulin resistance, we measured Akt phosphorylation (pAkt) following insulin stimulation. While control cells showed a strong increase in the pAkt/Akt ratio, this response was blunted in IR cells (Figure 2C). Downstream of pAkt, insulin regulates myocardial glucose metabolism. The glycolysis pathway was negatively enriched in the IR model, along with significantly lower expression of multiple glycolytic enzymes (Figure 2D-F, Figure S3A). We validated this with functional measurements of metabolic flux, finding that both insulin-stimulated glycolytic rates and total glucose metabolism were significantly lower in IR cardiomyocytes (Figure 2G, H).

### The 2D IR model demonstrates a metabolic shift towards increased fatty acid utilisation and changes in mitochondrial oxidative phosphorylation

**Figure 3:**
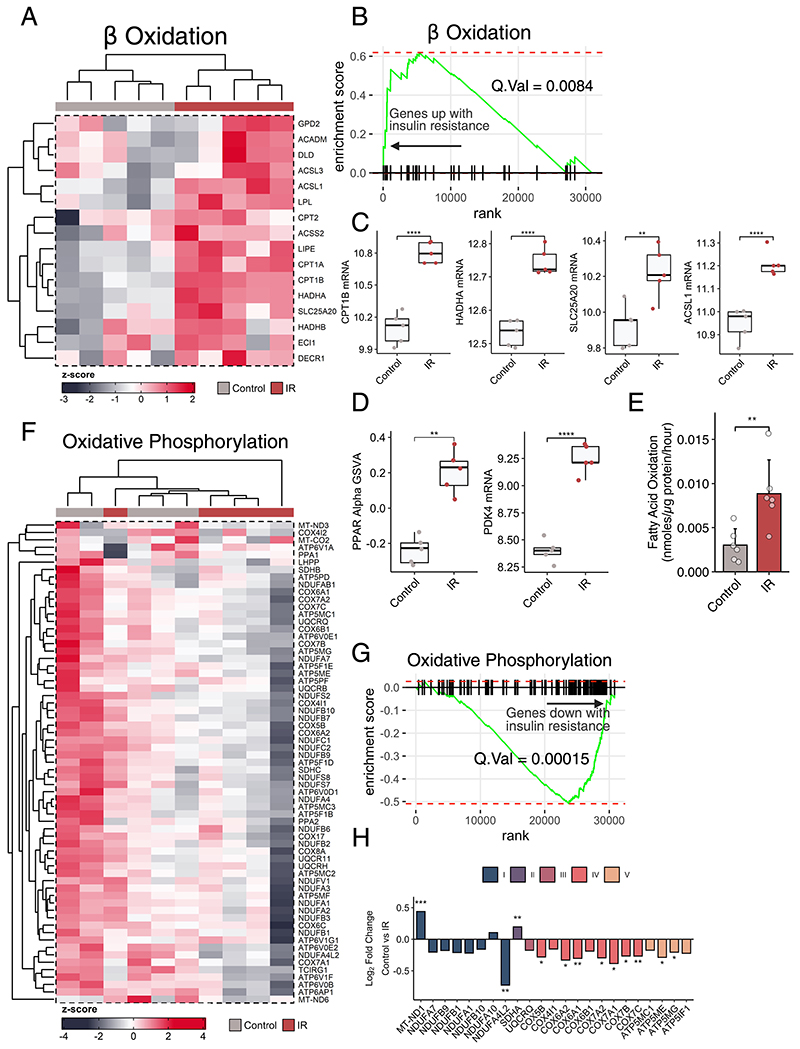
The 2D IR model demonstrates a metabolic shift towards increased fatty acid metabolism and changes in mitochondrial oxidative phosphorylation. (A-C) The mRNA expression of fatty acid oxidation pathway genes was elevated in insulin-resistant (IR) hiPSC-CMs. This is visualised at the pathway level (A, B; MsigDB: WikiPaths) and for the specific rate-limiting enzymes: carnitine palmitoyltransferase 1B (CPT1B), hydroxyacyl-CoA dehydrogenase A (HADHA), carnitine/acylcarnitine translocase (SLC25A20), and long-chain acyl-CoA synthetase 1 (ACSL1) (C). (D) Gene targets of the fatty acid transcription factor PPARα, including its downstream target pyruvate dehydrogenase kinase 4 (PDK4), were also significantly upregulated in IR hiPSC-CMs. Boxplots show median ± IQR; statistical analysis was performed using a Student’s t-test (D, left) or DESeq2 (C, D right) (*p < 0.05; **p < 0.01; ***p < 0.001; *n* = 5 samples per condition). (E) A bar plot of radioisotope flux measurements confirmed significantly elevated fatty acid oxidation rates in IR hiPSC-CMs compared with controls (Student’s t-test, **p < 0.01, *n* = 6). (F, G) Despite negative enrichment of the oxidative phosphorylation pathway (MsigDB: Kegg), the expression of leading-edge genes did not show robust group separation following hierarchical clustering. (H) The expression of most mitochondrial genes were significantly reduced in IR hiPSC-CMs, except for SDHA and MT-ND1, which showed increased expression compared with controls (log_2_ fold changes for control vs. IR; DESeq2, *p < 0.05; **p < 0.01; ***p < 0.001, *n* = 5).

In contrast to glycolysis, FA metabolism was significantly upregulated in our IR model. The β-oxidation pathway was positively enriched, and the expression of its leading-edge genes, including several rate-limiting enzymes, robustly separated the treatment groups (Figure 3A-C). Furthermore, target genes of the master FA metabolic regulator PPARα were collectively upregulated (Figure 3D, Figure S3B). Functional validation confirmed this metabolic reprogramming: FA oxidation rates were 2.9-fold higher in the IR group compared with controls (Figure 3E).

In DbCM, this reliance on FA is associated with mitochondrial dysfunction, and we found the OxPhos pathway to be negatively enriched in our model. While this did not result in robust clustering of the treatment groups (Figure 3F-G), several electron transport chain subunits demonstrated significantly altered expression levels (Figure 3H). Among those significantly changing, all except succinate dehydrogenase enzyme complex (SDHA) and mitochondrially-encoded NADH dehydrogenase 1 (MT-ND1) were negatively expressed in our IR model.

### Culturing 2D hiPSC-CMs in IR media blunts the response to hypoxia

**Figure 4:**
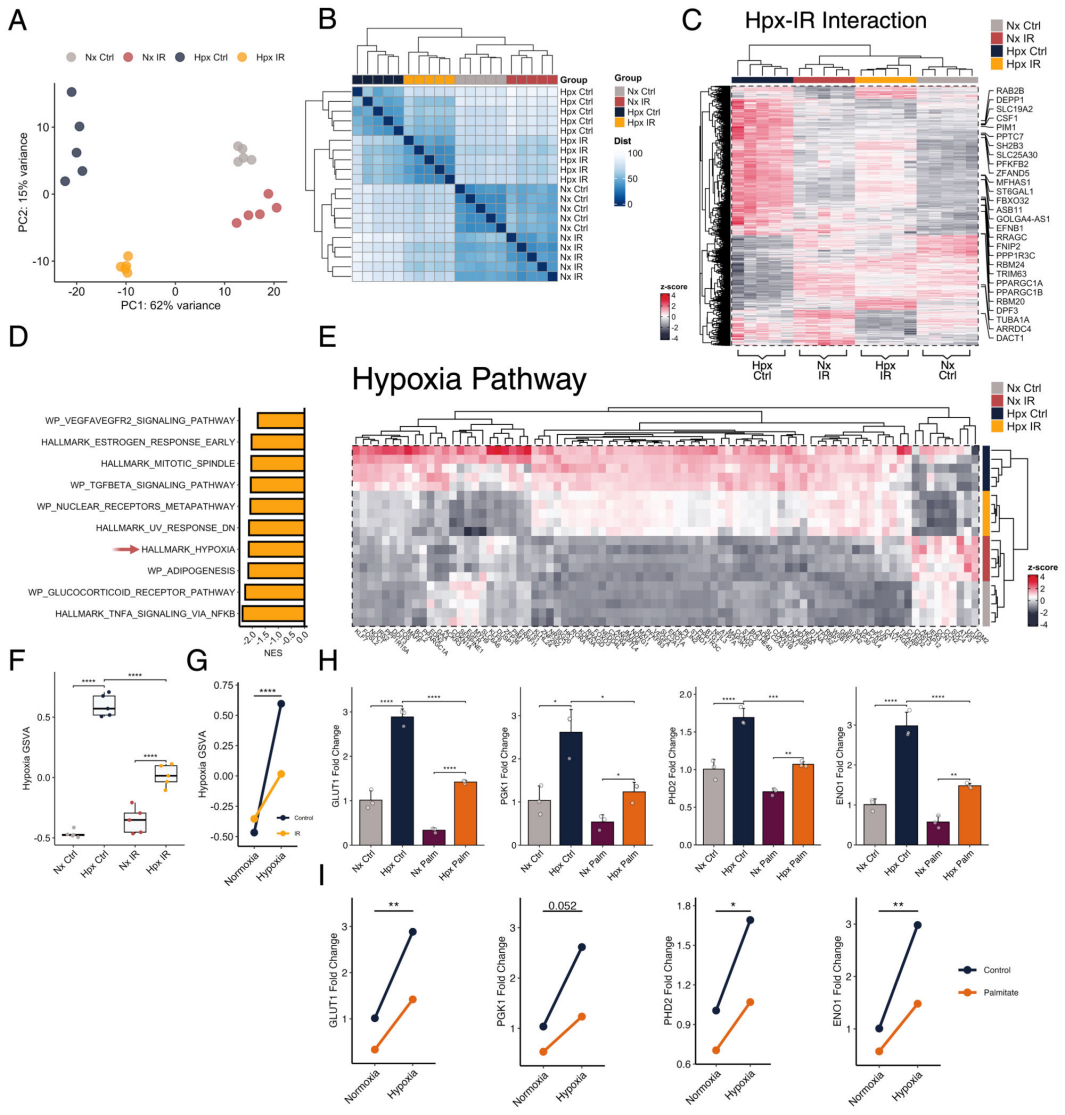
Culturing 2D hiPSC-CMs in IR media blunts the response to hypoxia. (A) Principal component analysis (PCA) of transcriptomic profiles from control and insulin-resistant (IR) hiPSC-CMs demonstrates distinct clustering by experimental condition (normoxia (Nx) vs. hypoxia (Hpx) and control (Ctrl) vs. IR (IR), *n* = 5). (B) Hierarchical clustering of sample distances shows that IR hypoxic samples cluster more closely with normoxic groups than with hypoxic controls. (C) Differential expression analysis identified genes with significant interaction effects between insulin resistance and oxygen status (DESeq2, FDR < 0.05). (D) Pathway analysis of genes ranked by their interaction effects reveals that hypoxia-related terms are prominent among the top ten significantly enriched pathways (FGSEA, FDR < 0.05). (E) Insulin resistance blunts the hypoxia-induced expression changes of leading-edge genes within the hypoxia pathway (MsigDB: Hallmark). (F) Boxplots (median ± IQR) of hypoxia pathway summary scores highlight a significantly reduced hypoxic response in IR samples. (G) Interaction plots further visualise this blunting effect (two-way ANOVA with Tukey’s post hoc test, ****p < 0.0001; *n* = 5). (H) Bar plots (mean ± SD) show that palmitate incubation is sufficient to blunt hypoxia-induced changes in the expression of target genes including glucose transporter 1 (GLUT1), phosphoglycerate kinase 1 (PGK1), prolyl hydroxylase domain 2 (PHD2), and enolase 1 (ENO1), as measured by RT-qPCR and normalised to UBC. Statistical analysis was performed using a two-way ANOVA with Tukey’s post hoc test. Due to non-normal distribution, PGK1 data were analysed using an Aligned Rank Transform (ART) ANOVA with post hoc comparisons. (*p < 0.05, **p < 0.01, ***p < 0.001, ****p < 0.0001; *n* = 3). (I) Interaction plots confirm the significant blunting effect of palmitate (two-way ANOVA, interaction *p < 0.05, **p < 0.01; *n* = 3).

The adaptive hypoxic response is impaired in patients with type 2 diabetes (T2DM) following myocardial infarction^41-43^, therefore, we investigated this in our model. While hypoxia induced a profound transcriptional shift in both control and IR cardiomyocytes, PCA and hierarchical clustering analyses revealed a severely blunted response in the IR group. Strikingly, hypoxic IR cells were transcriptionally more similar to normoxic cells than to hypoxic controls (Figure 4A-B).

An interaction analysis identified 3,034 genes whose response to hypoxia was specifically altered by the IR phenotype. For most of these genes, the IR condition attenuated their normal induction by hypoxia, leading to a significant suppression of core hypoxia signalling pathways (Figure 4C-E). Gene set variation analysis (GSVA) quantified this effect, confirming that while both groups responded to hypoxia, the magnitude of this response was significantly blunted in IR cardiomyocytes (Figure 4F-G). To identify the driver of this impaired response, we tested the individual components of the IR media. Culturing cardiomyocytes with palmitate alone was sufficient to recapitulate the blunted hypoxic signalling (Figure 4H-I). These data suggest that the lipid component of the diabetic milieu is the primary driver of impaired cardiac hypoxic adaptation.

### Signatures of cardiac stress and altered splicing in 2D, yet contractile changes were confounded by heterogeneity

Our IR model also exhibited hallmarks of cardiac stress, with significant enrichment of both TGF-β and ER stress pathways (Figure S4). Paradoxically, contractile gene sets were upregulated, prompting us to investigate post-transcriptional regulation (Figure S5A). This revealed 314 significant differential transcript usage (DTU) events concentrated in genes for myocardial structure and contraction, including the tropomyosin family (Figure S5B-D). These splicing changes coincided with the upregulation of the master cardiac splicing regulators RBM20 and RBM24, indicating a mechanism for generating altered contractile protein isoforms (Figure S5E). Despite this extensive molecular remodelling, direct contractility assays in our 2D model revealed no significant differences in contraction or relaxation dynamics, a result confounded by high experimental variability (Figure S5F-L). This suggests that while our model recapitulates key transcriptional features of DbCM, our 2D culture system may lack the structural organisation and mechanical load required to present the functional consequences of these molecular changes.

### Integrated transcriptomics and proteomics demonstrate network hubs in 3D-engineered heart tissue (EHT) that capture the disease phenotype

**Figure 5:**
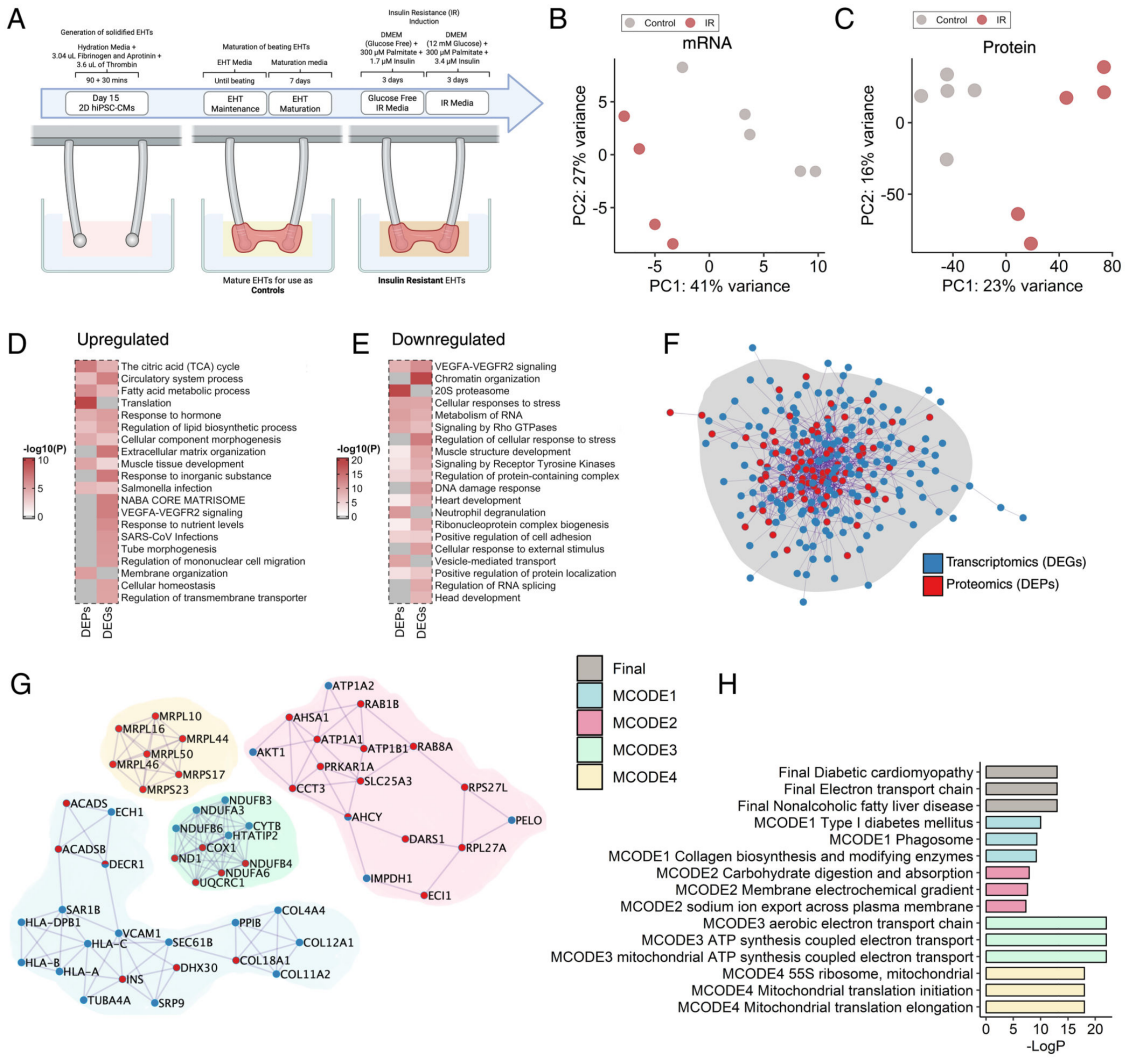
A 3D engineered heart tissue (EHT) model of insulin resistance captures features of diabetic cardiomyopathy. (A) Schematic showing the generation of engineered heart tissues (EHTs) by embedding hiPSC-CMs in fibrin-based hydrogels anchored within agarose moulds. (B,C) Principaln component analysis (PCA) of the transcriptomic (B) and proteomic (C) profiles demonstrates a clear separation between control and IR EHTs (transcriptomics: *n* = 5 control, *n* = 4 IR; proteomics: *n* = 5 per condition). (D, E) Pathway analysis of positively (D) and negatively (E) regulated, differentially expressed genes (DEGs) and proteins (DEPs) highlights an overrepresentation of metabolic pathways. (F, G) Protein-protein interaction networks (F) and their dense subnetworks (G), constructed from upregulated DEGs and DEPs in IR EHTs, reveal key associations with diabetic cardiomyopathy. (H) Bar plots showing the overrepresentation significance of the top three pathways within the subnetworks. The colours correspond to the pathway annotations and network shading.

Culturing hiPSC-CMs as 3D EHTs more closely replicates the increased workload and shear stress of *in situ* cardiomyocytes, to better recapitulate the mechanical environment of the heart (Figure 5A). Following exposure of EHT to IR-inducing media, we performed parallel transcriptomic and proteomic analyses. PCA of both datasets revealed a clear separation between IR and control EHTs (Figure 5B-C). We identified 321 downregulated and 224 upregulated genes (FDR-corrected), but only 11 proteins were significantly altered post-correction, prompting us to include proteins with a p-value < 0.05 (132 downregulated, 109 upregulated) for subsequent analysis. To overcome this limitation and reduce dimensionality, we integrated the transcriptomic data to prioritise biologically significant clusters via pathway and protein-protein interaction (PPI) analyses. Pathway enrichment analysis of upregulated molecules revealed over-representation for FA metabolism and ECM remodelling pathways (Figure 5D). Conversely, downregulated terms related to receptor tyrosine kinase signalling (the family for the insulin receptor) and vesicle-mediated transport (involved in glucose uptake) (Figure 5E). A PPI network integrating these differentially expressed molecules (Figure 5F-G) highlighted that the DbCM pathway and related metabolic pathways were significantly over-represented (Figure 5H).

### The IR EHT model demonstrates a metabolic shift from glucose to fatty acids, accompanied by contractile dysfunction

**Figure 6:**
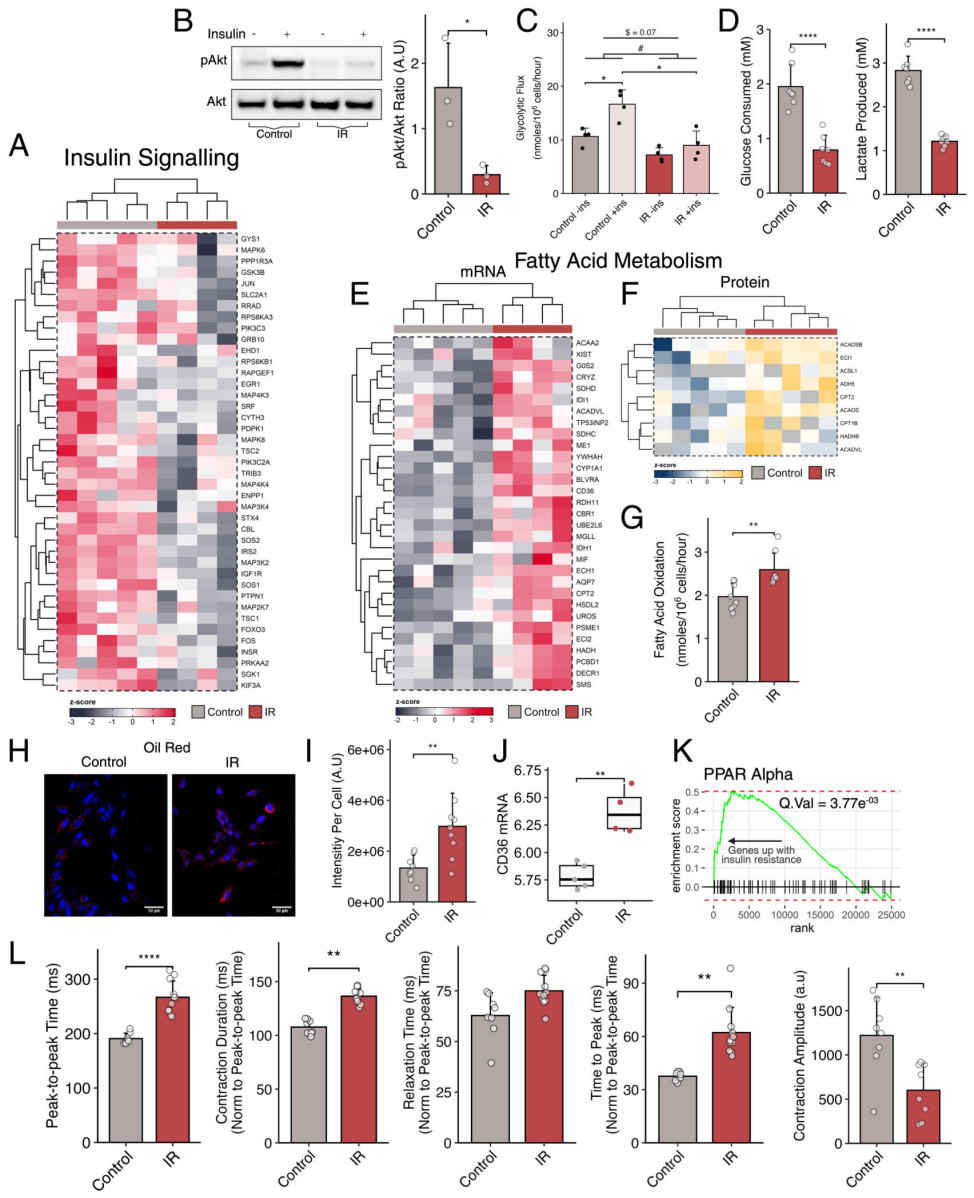
The IR EHT model demonstrates a metabolic shift from glucose to fatty acids accompanied by contractile dysfunction. (A) Blunted insulin signalling in IR EHTs was visualised by reduced expression of leading-edge genes for the significantly negatively enriched insulin signalling pathway (MsigDB: WikiPaths; FGSEA, FDR < 0.05). (B, left) Western blots showed phosphorylated (pAkt) and total Akt in control and IR EHTs, with the quantified pAkt/total Akt ratio (B, right, mean ± SD) showing a significant reduction in IR compared with controls (Student’s t-test, *p < 0.05, *n* = 3 EHT per condition). (C) Glycolytic flux measured using radioisotopes with and without insulin (ins) stimulation, was significantly reduced in IR EHTs compared with controls (mean ± SD; two-way ANOVA with Tukey’s post hoc test, *p < 0.05; #*p* < 0.05 main effect of IR; $*p* = interaction term; *n* = 4). (D) Barplots (mean ± SD) show change in glucose metabolism and lactate production in cell culture media from 1 EHT over 24 hours (Student’s t-test, ****p < 0.0001, *n* = 7). (E, F) Fatty acid metabolism was upregulated in IR EHTs, as shown by the expression of leading-edge genes (E) and proteins (F) for the positively enriched fatty acid metabolism pathway (MSigDB: Hallmark; FGSEA, FDR < 0.05). (G) Elevated fatty acid oxidation rates in IR EHTs were confirmed by radioisotope flux measurements (mean ± SD; *p* < 0.01, Wilcoxon rank sum, *n* = 8). (H, I) Representative Oil Red O staining (H) and quantified lipid droplet accumulation (I) revealed significant lipid storage in IR EHTs (mean ± SD, Student’s t-test, **p < 0.01, control *n* = 8, IR *n* = 9). (J) CD36 expression (median IQR; DESeq FDR *p < 0.05, control *n* = 5, IR *n* = 4) was significantly increased in IR EHTs. (K) The PPARα signalling pathway was positively enriched in IR samples (FGSEA). (L) MUSCLEMOTION contractility analysis (mean ± SD; *n* = 8-11). Cycle length (peak-to-peak) and contraction amplitude were analysed by Student’s t-test (**p < 0.01, ****p < 0.0001). Contraction duration, relaxation time, and time-to-peak were rate-corrected via ANCOVA (*Log-metric ~ Media + Log cycle length*) by adjusting to a common cycle length (231 ms) for group comparison (**p < 0.01). Bars show raw (t-test) or adjusted (ANCOVA) means; dots are biological replicates (*n* = 8-11).

Aligning with our monolayer findings, the insulin signalling pathway was significantly repressed (NES = -1.49, FDR = 0.037), and samples clustered distinctly by treatment (Figure 6A). This was functionally confirmed by an 80% reduction in the p-Akt/Akt ratio upon insulin stimulation and a 46% decrease in glycolytic flux in IR EHTs (Figure 6B-C). In agreement, both glucose metabolism and lactate production significantly decreased with IR (Figure 6D). Concurrently, the FA metabolism pathway was positively enriched at both the transcriptomic (NES = 1.78, FDR = 0.00093) and proteomic levels (NES = 1.75, *p* = 0.0093), again robustly clustering the samples (Figure 6E, F). This culminated in a 32% increase in FA oxidation rates (Figure 6G) and a two-fold increase in intracellular lipid accumulation (Figure 6H-I), indicating a pathological shift not observed with maturation alone (Figure S1A, B). This was associated with positive enrichment of the PPARα signalling pathway, including the sarcolemmal FA transporter CD36 (Figure 6J-K). Critically, this 3D model allowed for functional contractile assessment, revealing that IR EHTs exhibited hallmarks of contractile dysfunction, including increased peak-to-peak time, contraction duration, time to peak, and decreased contraction amplitude compared with controls (Figure 6L). Relaxation time was significantly longer in IR than in control when analysed without rate adjustment (Figure S6A).

### Comparison of 2D and 3D models of DbCM revealed conserved changes in contraction-related pathways, but only 3D translated to changes in contractility

**Figure 7:**
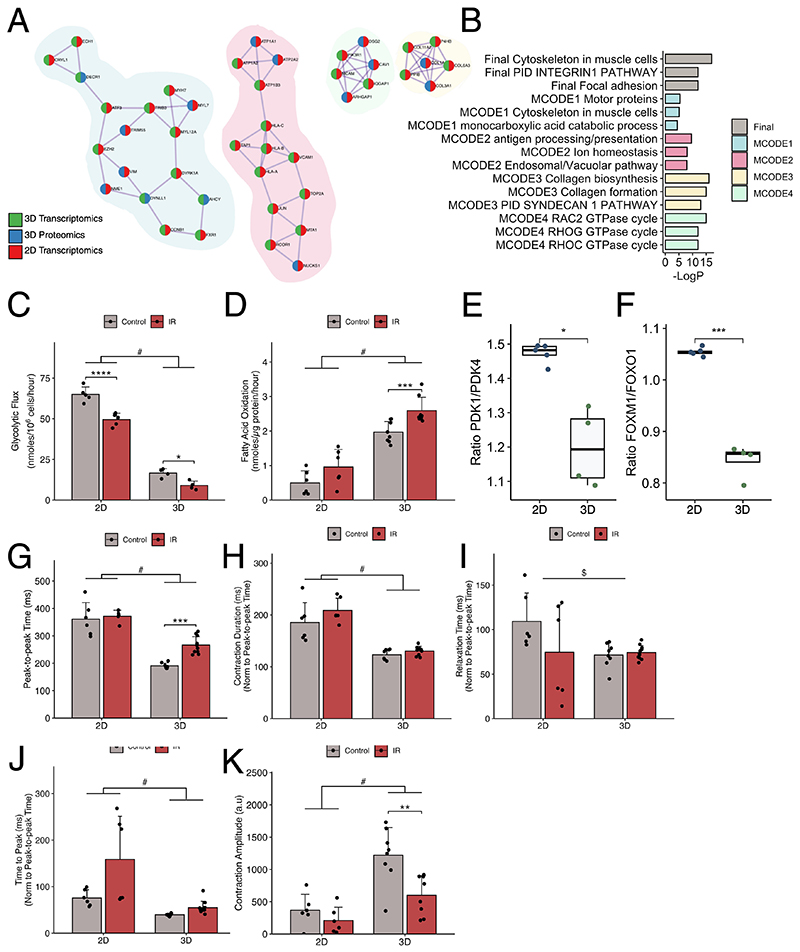
‘Omics highlights contractile dysfunction as a core feature in both IR models, with physiological measurements aligning in the 3D IR system. (A) An integrated network and its dense subnetworks were constructed from differentially expressed genes (in 2D and 3D) and proteins (in 3D) under insulin-resistant conditions, based on known protein-protein interactions. (B) Bar plots show the overrepresentation significance for the top three pathways within the subnetworks, with colours corresponding to pathway annotations and network shading. (C, D) Barplots show glycolytic (C, 2D *n* = 5, 3D *n* = 4) and fatty acid oxidation (D, 2D *n* = 6, 3D *n* = 8) flux measurements from 2D and EHT models under control and IR conditions (mean ± SD). (C) was analysed by two-way ANOVA with post-hoc Tukey comparisons. (D) was analysed by Aligned Rank Transform (ART) ANOVA with post-hoc comparisons (***p < 0.001, ****p < 0.0001 for post-hoc comparisons, # *p* < 0.05 for main tissue effect). (E, F) Boxplots (median ± IQR) displaying normalised transcriptomic expression ratios of key genes for IR 2D hiPSC-CMs and 3D EHTs (Student’s t-test, *p < 0.05, ***p < 0.001; *n* = 5 wells for 2D, *n* = 4 EHTs for 3D). (G–K) Contractility metrics from 2D and 3D models. (G, K) Cycle length (peak-to-peak time) and contraction amplitude (2D *n* = 6, 3D *n* = 8) were directly analysed by two-way ANOVA with Tukey post-hoc (mean ± SD; **p < 0.01, ***p < 0.001 for indicated comparisons). (H–J) The other metrics—contraction duration, relaxation time, and time-to-peak—were rate-corrected by adjusting to a common cycle length (268 ms) using a random-slope mixed model: *log(metric) ~ Tissue × Media + log(Peak-to-peak) + (1 + log(Peak-to-peak)* | *Tissue:Media)*. Bars for (G) and (K) show mean ± SD, while bars for (H-J) show the adjusted mean ± SD. Dots are individual replicates (2D *n* = 6; 3D Control *n* = 8, 3D IR *n* = 10-11). Significance codes: # 2D vs 3D effect (Tissue main term, *p* < 0.05), $ interaction term (Tissue × Media, *p* < 0.05).

To compare the pathological response of the 2D and 3D models, we performed an integrated pathway analysis. While distinct technologies precluded direct ‘omics comparisons, over-representation analysis of DEGs and DEPs identified shared IR-induced pathways, including cardiac contraction, with common targets including TRIB3, PIK3R1, and DECR1 (Figure 7A-B). Functionally, both models exhibited a pathological shift from glycolytic to FA metabolism under IR conditions. However, EHTs displayed inherently lower glycolytic and higher FA metabolic rates, confirming their advanced metabolic maturity (Figure 7C-D). This enhanced oxidative metabolism in EHTs coincided with lower PDK1/PDK4 and FOXM1/FOXO1 gene expression ratios, key regulators of metabolic substrate preference (Figure 7E-F).

Crucially, while the IR-induced contractile dysfunction was only significant in 3D EHTs, direct comparison of the platforms revealed significant baseline differences, with culture dimension having a main effect on peak-to-peak time, contraction duration, time to peak, and contraction amplitude (Figure 7G, H, J, K). Furthermore, a significant interaction effect was observed for relaxation time; for instance, IR increased relaxation time in EHTs while decreasing it in 2D cultures (Figure 7I). This functional deficit in IR EHTs was underpinned by structural changes; analysis of the well-defined sarcomeres (Figure S6B-F) revealed that IR induced a significant reduction in muscle fibre area relative to cell area.

### Metformin Rescues the Metabolic and Contractile Dysfunction in IR EHTs

**Figure 8:**
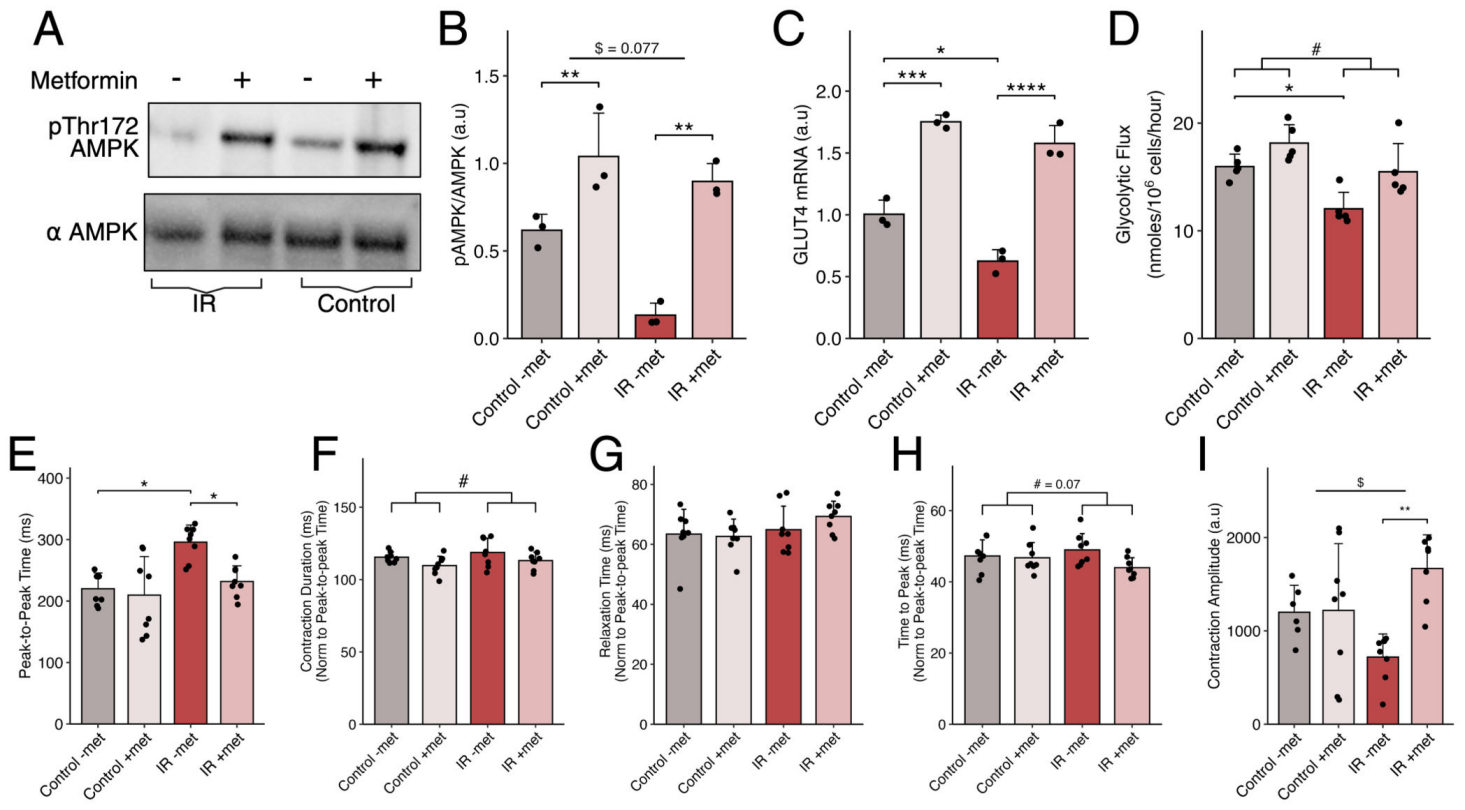
Metformin Rescues the Metabolic and Contractile Dysfunction in IR EHTs. (A) Representative western blots show phosphorylated AMPK (pAMPK, Thr172) and total AMPK levels in insulin-resistant (IR) and control engineered heart tissues (EHTs), with or without metformin (met) treatment. (B-D) Bar plots (mean ± SD) quantify the effects of metformin across conditions, analysed by two-way ANOVA with Tukey's post-hoc comparisons, showing the ratio of pAMPK to total AMPK (B, *n* = 3), GLUT4 expression measured by RT-qPCR and normalised to UBC (C, *n* = 3), and glycolytic flux (D, *n* = 5) (*p < 0.05, **p < 0.01, ***p < 0.001, ****p < 0.0001, # main effect of metformin treatment < 0.05, $ interaction effect < 0.05). B and D were not normally distributed and were first transformed with aligned ranks (ART ANOVA). (E-I) Contractility was also analysed in IR and control EHTs, with and without metformin treatment. (E, I) Cycle length (peak-to-peak time) and contraction amplitude are shown as mean ± SD and were analysed by two-way ANOVA with Tukey's post-hoc test (*p < 0.05, *n* = 6-8). (F-H) The other contractile metrics—contraction duration, relaxation time, and time-to-peak—were rate-corrected by adjusting to a common cycle length (232 ms) using a random-slope mixed model: log(metric) ~ Treatment * Media + log(Peak-to-peak) + (1 + log(Peak-to-peak) | *Treatment:Media)*. For panels F-H, bars show the adjusted values (mean ± SD). Dots in all panels represent individual biological replicates. The significance codes are: # indicates a significant main effect of metformin treatment (*p* < 0.05), and $ indicates a significant interaction effect (*p* < 0.05).

To validate the IR EHT model for therapeutic screening we tested the effects of metformin, and found it successfully rescued molecular and functional deficits induced by IR. Metformin restored AMP-activated protein kinase (AMPK) phosphorylation, which was significantly reduced by IR, back to control levels (Figure 8A-B). This rescue of AMPK activity corresponded with a normalisation of GLUT4 expression and restoration of glycolytic flux rates, reversing the glucose metabolic dysfunction induced by IR (Figure 8C-D). Metformin also corrected the IR-induced contractile dysfunction. It significantly mitigated the prolonged peak-to-peak time and reduced contraction amplitude (Figure 8E, I) and exerted a significant main treatment effect on contraction duration (Figure 8F-G). The significant interaction effect for contraction amplitude, in particular, suggests a specific reversal of the IR-induced contractile deficit. Taken together this underscores the potential value of the IR EHT platform for evaluating therapies for diabetic cardiomyopathy.

## Discussion

Human-centric models of diabetic cardiomyopathy (DbCM) are crucial for understanding disease mechanisms and developing new therapies. We generated a DbCM model by culturing hiPSC-CM in 2D or 3D in “IR” media, and applied a systems biology approach to assess affected pathways. Our model activated key pathological pathways, including a shift from glucose to FA metabolism, mitochondrial dysfunction, ECM remodelling, and ER stress. Crucially, the 3D EHT IR model further recapitulated contractile dysfunction, a clinical hallmark of the disease. Contractile and metabolic impairments were pharmacologically reversible with metformin, validating this human iPSC-CM platform as a potential tool for investigating DbCM pathophysiology and testing therapeutic interventions.

### Culturing cells in IR media induces a transcriptional profile identified as Diabetes Mellitus

While hiPSC-CMs are well-established for modelling monogenic disorders, their applications to complex, systemic diseases have been limited to single-substrate protocols, short exposure times, or immature hiPSC-CMs^15-19^. To address this, we cultured hiPSC-CMs in a bespoke IR media that mirrors the chronic hyperglycaemia, hyperlipidaemia, and hyperinsulinaemia of T2DM. ‘Omic analysis demonstrated induction of a disease-relevant state: enriched for ‘Diabetes Mellitus, Experimental’ and ‘Diabetic cardiomyopathy’ pathways, and known human DbCM biomarkers, such as natriuretic peptide A (electrolyte homeostasis), nebulin (cytoskeleton), and frizzled-related protein (Wnt signalling)^40^, robustly clustered control and IR samples.

### 2D and 3D IR hiPSC-CMs demonstrate the metabolic shift from glucose to fatty acid utilisation

A hallmark of the T2DM heart is the metabolic shift from glucose to FA utilisation^4^, and our IR models recapitulated this in both 2D and 3D formats. Increased FA metabolism was evident in transcriptomic and proteomic profiles—likely driven by PPARα activation^44^—and was functionally validated by radioisotope flux measurements showing increased FA oxidation and concomitantly decreased glucose metabolism. Additionally, upregulated lipid biosynthesis pathways led to enhanced triglyceride accumulation and activation of ceramide-related pathways linked to IR. Critically, a prior maturation step with low oleate concentrations established metabolic readiness in our cells^13^. We propose that pre-conditioning was essential for mitigating apoptosis during the IR protocol by priming cells to effectively metabolise the high lipid load.

### The IR model impacts key mediators implicated in the development of DbCM

DbCM is marked by contractile dysfunction and fibrosis, driven by mediators including ER stress and ECM remodelling^4^, and our omics profiling revealed upregulation of genes for these pathways. In 3D EHTs, proteins and genes linked to ECM remodelling and collagen biosynthesis formed a dense subnetwork, aligning with diabetes-associated fibroblast activation^45^. The likely presence of hiPSC-fibroblasts in our cultures is therefore potentially advantageous for studying the fibrotic component of DbCM. Contractile dysfunction in DbCM stems from interconnected cellular abnormalities^46^, and we identified several significant genes/pathways consistent with impaired actin-myosin cross-bridge cycling via myosin heavy chain alterations^47^; energy deficits limiting ATP for actomyosin interaction and ion pumps^48^; dysregulated ion channels disrupting calcium homeostasis and excitation-contraction coupling^49^; and cytoskeletal remodelling via GTPases like RhoA, which, with excessive collagen deposition, increase myocardial stiffness^50,51^.

### Advantages and disadvantages of modelling IR in 2D vs. 3D

Our results show a key distinction between platforms: while both 2D and 3D IR models exhibited transcriptional and metabolic changes, only the 3D EHT model recapitulated contractile dysfunction including longer contraction duration and peak amplitude, as well as increased relaxation time. The superior physiological fidelity of the 3D EHT platform is rooted in its promotion of a more mature cardiomyocyte phenotype via mechanical strain and complex cell-matrix interactions absent in monolayers^22^. However, this does not discount 2D monolayers, which are well-suited for high-throughput target identification for development of treatments for DbCM. For a detailed assessment of integrated pathophysiology, however, their shortcomings are clear. Future work that paces cells for more precise contraction analysis may further reduce the heterogeneity in these measurements.

Our objective was not to perfectly replicate a systemic pathology like DbCM, but to engineer a 'fit-for-purpose' iPSC-cardiomyocyte system for probing the cell-intrinsic consequences of DbCM. We acknowledge that our protocol does not fully replicate the multifactorial nature of chronic T2DM, however, the modularity of this hiPSC-CM platform provides a powerful framework for dissecting pathogenic factors. The IR media could be further supplemented with pro-inflammatory cytokines to model inflammation-driven fibrosis, advanced glycation end-products to recapitulate myocardial stiffening, or intermittent hypoxia to simulate microvascular insufficiency^52^. Additionally, co-culture with iPSC-derived cardiac fibroblasts or immune cells would allow the model to evolve to include the multiple cell types implicated in DbCM^53^. Systematically adding these layers of complexity will enhance the model's predictive power for dissecting disease mechanisms and identifying novel therapeutic targets.

Given that up to 70% of patients with type 2 diabetes exhibit DbCM^5,54^ amid limited treatment options^6,7^, we evaluated our EHT model’s response to metformin therapy. We demonstrate that metformin restored glycolytic metabolism and concurrently alleviated contractile dysfunction in our IR-EHTs, confirming the model’s suitability for assessing the direct effects of therapeutics on the cardiomyocyte.

In conclusion, our multi-factorial IR protocol, applied to metabolically mature hiPSC-CMs, induced key mechanisms of DbCM that were confirmed by a systems biology approach. The 3D EHTs further recapitulated a contractile dysfunction phenotype that was demonstrably reversed by metformin, underscoring the platform's value for therapeutic screening.

## Supplementary Material

Supplementary Materials

## Data Availability

The datasets generated during and/or analyzed during the current study are available from the corresponding author upon reasonable request

## Highlights

- Human-centric cardiac models are needed that recapitulate mechanistic and functional changes in the type 2 diabetic myocardium, for understanding disease pathogenesis and developing new therapies.
- Using human iPSC-derived cardiomyocytes in 2D and 3D, as engineered heart tissue (EHT), we aimed to model diabetic cardiomyopathy *in cellulo*.
- Taking an unbiased systems biology approach, our cellular models recapitulated the dysregulated pathways and functional derangement of diabetic cardiomyopathy. 3D EHT models showed contractile dysfunction akin to that seen in patients, with mechanistic and functional changes reversed with metformin.
- Therefore, it is possible to generate translationally-relevant human iPSC-derived cardiomyocyte models that mimic diabetic cardiomyopathy.
