# Supplementary material for: Systems biology and functional assessments of human iPSC-cardiomyocyte models of insulin resistance captures key hallmarks of diabetic cardiomyopathy
Source: Diabetes. Author manuscript; Available in PMC 2026 May 15. (PMC12585165; doi:10.2337/db25-0204)
Supplement: Supplementary Materials [file EMS209518-supplement-Supplementary_Materials.pdf]

Supplementary Table.

Supplementary Table 1: qPCR human primer sequences

**Target**	**Forward and Reverse primer sequences**
GLUT1	Fp: AAGCTGACGGGTCGCCTCATG<br>Rp: CTCTCCCCATAGCGGTGGACC
PGK1	Fp: GAATGGGAAGCTTTTGCCCG<br>Rp: GCAGTGTCTCCACCACCTATG
PHD2	Fp: CAGAAGGCAAAGCCCAGTTTG<br>Rp: CCTCACACCTTTTTCACCTTGTAG
ENO1	Fp: GCCCTGGTTAGCAAGAAACT<br>Rp: GAATGGCGTTCGCACCAAAC
UBC	Fp: CCTGGTGCTCCGTCTTAGAG<br>Rp: TTTCCCAGCAAAGATCAACC

Supplementary Table 2: Antibody details

Antibody	Supplier
pAkt	Abcam (Ab81283)
Akt	Abcam (Ab32505)
pAMPK (Thr 172)	Cell Signaling (2535)
AMPKα	Cell Signaling (2603)
Anti-rabbit HRP secondary antibody	Abcam (Ab205718)

Supplementary Figures

Figure S1 – Cell death and apoptosis were not significantly induced by culturing in IR media. (A) Representative Oil Red O staining for immature (left) and mature (right) 2D hiPSC-CMs. (B) Quantified lipid droplet accumulation (mean ± SD, n = 3) reveals a trend for reduced lipid storage with maturity (Student's *t*-test). (C) Fluorescence microscopy of live/dead staining shows limited induction of cell death with the insulin-resistance (IR) protocol after 3 days and 6 days, in 2D hiPSC-CMs. Live/dead staining was carried out using Readyprobes® consisting of NucBlue® Live reagent and NucGreen® Dead reagent, and cells were imaged using fluorescence microscopy using the DAPI and GFP filters, respectively (D, E) On a pathway level, the apoptosis pathway was negatively enriched for IR 2D hiPSC-CMs (D, FGSEA) and showed negative expression with IR for related changes (E).

Figure S2 – Tissue enrichment analysis reveals enrichment for 'heart muscle' in control and IR 2D transcriptomic samples. The bar graphs (top panels) displays the statistical significance of tissue enrichment for the list of expressed genes from control (A) and IR (B) samples. The TissueEnrich algorithm uses a hypergeometric test to calculate the over-representation of the input gene list within its curated, tissue-specific gene sets. The bottom panel shows the specific genes from the input list that are members of the 'Heart Muscle' gene set within the TissueEnrich database. This provides a detailed view of the individual cardiac-specific genes that are driving the significant 'Heart Muscle' enrichment score shown in the top panel

Figure S3 – Pathview visualisation of gene expression changes between control and IR 2D cells using the KEGG glycolysis and PPAR alpha pathways. (A, B) KEGG pathway maps for Glycolysis and PPARα signalling were generated using the Pathview package. Genes are coloured according to their DESeq2-derived $\log_2$ fold change (logFC) when comparing IR to control samples, where red signifies upregulation and green signifies downregulation with IR.

Figure S4 – Fibrosis and ER stress-related pathways were significantly enriched in IR cells. (A, B) mRNA expression of TGF beta pathway genes (MsigDB: WikiPaths) was significantly up-regulated in IR hiPSC-CMs (A) and visualised at the pathway level (B). (C, D) mRNA expression of ER stress pathway genes (MsigDB: WikiPaths) was significantly up-regulated in IR hiPSC-CMs (C) and visualised at the pathway level (D). (B, D) Heatmaps show z-score DESeq2 normalised expression values arranged by hierarchical clustering.

Figure S5 – Despite significant changes in expression and splicing for contraction-related genes, 2D hiPSC-CMs in IR media did not show altered contractile measures. (A) The cardiac muscle contraction pathway (MsigDB: KEGG) was significantly and positively enriched (GSEA FDR < 0.01) in IR cells, and visualised as a heatmap for the z-score normalised expression values. (B) Differential transcript usage (DTU) was performed to identify splicing variants. The volcano plot depicts DTUs significantly changing below < 0.05 FDR, with the labels depicting select contractile and insulin resistance-related genes. (C) The dot plot shows the p-values for the top pathways significantly overrepresented in the DTUs, which include several contraction-related terms. (D) Barplots depict proportional changes in transcripts for the genes tropomyosin (TPM) 3 and 2. (E) Boxplots showing significantly increased expression for RNA binding motif protein (RBM) 20 and RBM24 genes (median ± IQR; DESeq2, ****p < 0.0001). (F) The contraction graph generated from MUSCLEMOTION indicates contraction

measures, which include time to peak, the time taken to reach peak amplitude; relaxation time, the time taken to return to baseline; contraction duration, the sum of time to peak and relaxation time; peak-to-peak, which measures the time between peak amplitudes, and peak amplitude. (G) Schematic representation of contraction parameters measured in cardiomyocytes. (H, L) Cycle length (peak-to-peak time) and contraction amplitude were directly compared using Student's t-tests (mean ± SD; $p > 0.05$). (I-K) The other contractile metrics—contraction duration, relaxation time, and time-to-peak—were rate-corrected by adjusting to a common cycle length (339 ms) using an ANCOVA. The model *(Log-metric ~ Media + Log cycle length)* provided the group comparison ($p > 0.05$). Bars for (H) and (L) show mean ± SD, while bars for (I-K) show the adjusted mean ± SD. Dots are individual biological replicates ($n = 6$).

Figure S6 - Electron microscopy reveals disrupted sarcomeres within IR EHTs. (A) Relaxation time in control and IR EHTs (mean ± SD; Student's *t*-test, $*p < 0.05$; Control $n = 8$, IR $n = 12$). (B, C) Representative electron microscopy (EM) images of control (B) and IR EHTs (C) highlight z-disc sarcomeric structures (arrows) with scale bars at 5 µm. (D, E) EM images of control (D) and IR EHTs (E) display mitochondria (green) and muscle fibres (red) annotated in ImageJ; scale bars = 2 µm. (F) Dot plots compare normalised muscle areas in control vs. IR EHTs, analysed via Wilcoxon rank sums ($*p < 0.05$, control 135 grids from n =3, IR 44 grids from n = 3).

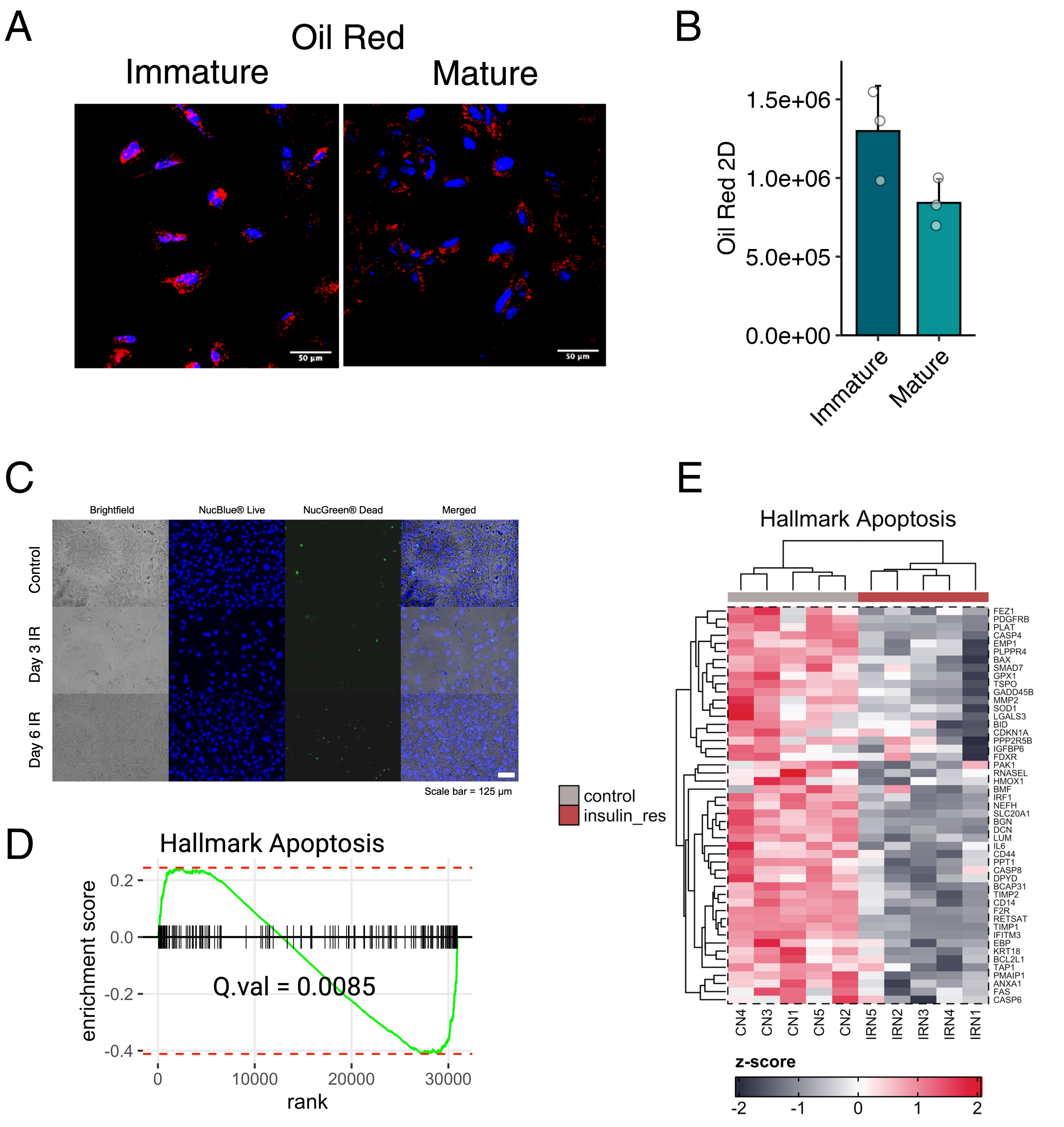
A
Oil Red
Immature
Mature
50 µm
50 µm
B
Oil Red 2D
1.5e+06
1.0e+06
5.0e+05
0.0e+00
Immature
Mature
C
Brightfield
NucBlue® Live
NucGreen® Dead
Merged
Control
Day 3 IR
Day 6 IR
Scale bar = 125 µm
D
Hallmark Apoptosis
enrichment score
0.2
0.0
-0.2
-0.4
Q.val = 0.0085
0
10000
20000
30000
rank
E
Hallmark Apoptosis
control
insulin_res
FEZ1
PDGFRB
PLAT
CASP4
EMP1
PLPPR4
BAX
SMAD7
GPX1
TSPO
GADD45B
MMP2
SOD1
LGALS3
BID
CDKN1A
PPP2R5B
IGFBP6
FDXR
PAK1
RNASEL
HMOX1
BMF
IRF1
NEFH
SLC20A1
BGN
DCN
LUM
IL6
CD44
PPT1
CASP8
DPYD
BCAP31
TIMP2
CD14
F2R
RETSAT
TIMP1
IFITM3
EBP
KRT18
BCL2L1
TAP1
PMAIP1
ANXA1
FAS
CASP6
CN4
CN3
CN1
CN5
CN2
IRN5
IRN2
IRN3
IRN4
IRN1
z-score
-2
-1
0
1
2

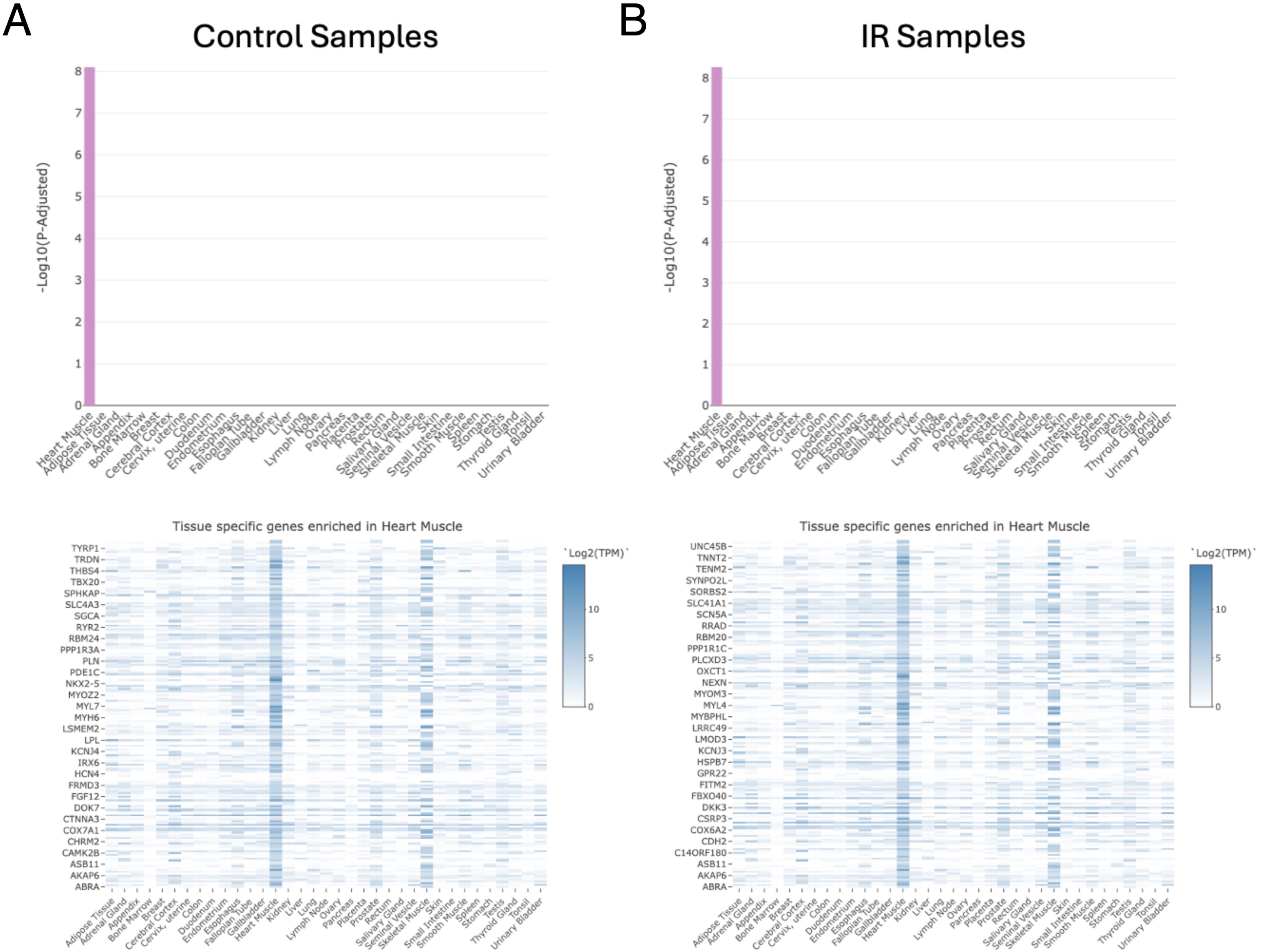
A
Control Samples
-Log10(P-Adjusted)
Heart Muscle
Adipose Tissue
Adrenal Gland
Appendix
Bone Marrow
Breast
Cerebral Cortex
Cervix, uterine
Colon
Duodenum
Endometrium
Esophagus
Fallopian Tube
Gallbladder
Kidney
Liver
Lung
Lymph Node
Ovary
Pancreas
Placenta
Prostate
Rectum
Salivary Gland
Seminal Vesicle
Skeletal Muscle
Skin
Small Intestine
Smooth Muscle
Spleen
Stomach
Testis
Thyroid Gland
Tonsil
Urinary Bladder
Tissue specific genes enriched in Heart Muscle
`Log2(TPM)`
TYRP1
TRDN
THBS4
TBX20
SPHKAP
SLC4A3
SGCA
RYR2
RBM24
PPP1R3A
PLN
PDE1C
NKX2-5
MYOZ2
MYL7
MYH6
LSMEM2
LPL
KCNJ4
IRX6
HCN4
FRMD3
FGF12
DOK7
CTNNA3
COX7A1
CHRM2
CAMK2B
ASB11
AKAP6
ABRA
B
IR Samples
UNC45B
TNNT2
TENM2
SYNPO2L
SORBS2
SLC41A1
SCN5A
RRAD
RBM20
PPP1R1C
PLCXD3
OXCT1
NEXN
MYOM3
MYL4
MYBPHL
LRRC49
LMOD3
KCNJ3
HSPB7
GPR22
FITM2
FBXO40
DKK3
CSRP3
COX6A2
CDH2
C14ORF180
ASB11
AKAP6
ABRA

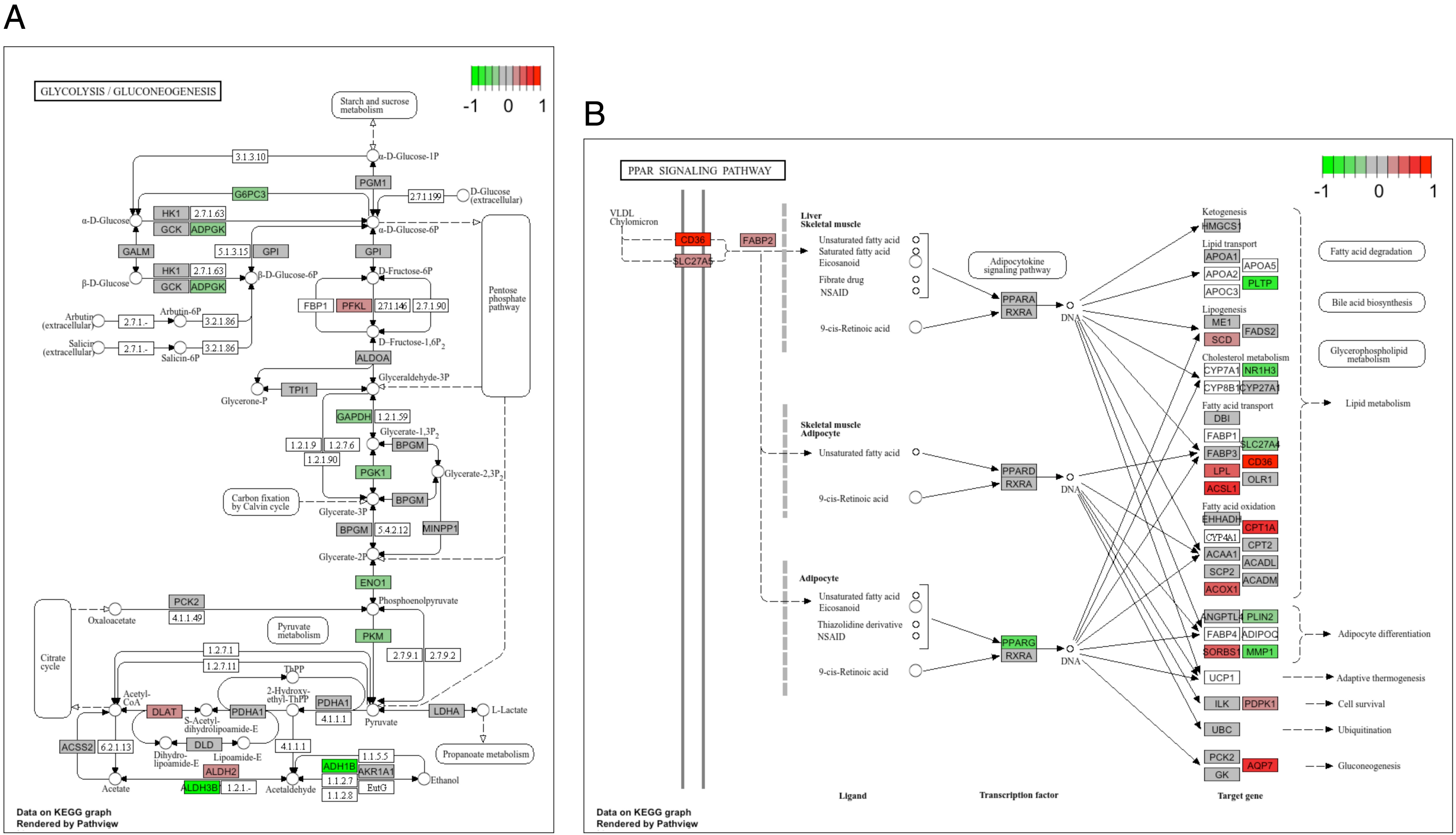
A
GLYCOLYSIS / GLUCONEOGENESIS
-1
0
1
Starch and sucrose metabolism
α-D-Glucose-1P
3.1.3.10
PGM1
G6PC3
2.7.1.199
D-Glucose (extracellular)
α-D-Glucose
HK1
2.7.1.63
GCK
ADPGK
α-D-Glucose-6P
GALM
5.1.3.15
GPI
β-D-Glucose
β-D-Glucose-6P
D-Fructose-6P
Pentose phosphate pathway
FBP1
PFKL
2.7.1.146
2.7.1.90
Arbutin (extracellular)
2.7.1.-
Arbutin-6P
3.2.1.86
Salicin (extracellular)
Salicin-6P
D-Fructose-1,6P2
ALDOA
Glyceraldehyde-3P
TPI1
Glycerone-P
GAPDH
1.2.1.59
Glycerate-1,3P2
1.2.1.9
1.2.7.6
BPGM
1.2.1.90
PGK1
Glycerate-2,3P2
Carbon fixation by Calvin cycle
Glycerate-3P
BPGM
5.4.2.12
MINPP1
Glycerate-2P
ENO1
PCK2
Phosphoenolpyruvate
Oxaloacetate
4.1.1.49
Citrate cycle
Pyruvate metabolism
PKM
1.2.7.1
2.7.9.1
2.7.9.2
1.2.7.11
ThPP
Acetyl-CoA
2-Hydroxy-ethyl-ThPP
DLAT
PDHA1
PDHA1
LDHA
L-Lactate
S-Acetyl-dihydrolipoamide-E
4.1.1.1
Pyruvate
ACSS2
6.2.1.13
DLD
4.1.1.1
Propanoate metabolism
Dihydro-lipoamide-E
Lipoamide-E
ADH1B
1.1.5.5
ALDH2
AKR1A1
Ethanol
Acetate
ALDH3B1
1.2.1.-
Acetaldehyde
1.1.2.7
1.1.2.8
EutG
Data on KEGG graph
Rendered by Pathview
B
PPAR SIGNALING PATHWAY
-1
0
1
VLDL
Chylomicron
CD36
SLC27A5
FABP2
Liver
Skeletal muscle
Unsaturated fatty acid
Saturated fatty acid
Eicosanoid
Fibrate drug
NSAID
9-cis-Retinoic acid
Adipocytokine signaling pathway
PPARA
RXRA
DNA
Skeletal muscle
Adipocyte
Unsaturated fatty acid
9-cis-Retinoic acid
PPARD
RXRA
DNA
Adipocyte
Unsaturated fatty acid
Eicosanoid
Thiazolidine derivative
NSAID
9-cis-Retinoic acid
PPARG
RXRA
DNA
Ketogenesis
HMGCS1
Lipid transport
APOA1
APOA5
APOA2
PLTP
APOC3
Lipogenesis
ME1
FADS2
SCD
Cholesterol metabolism
CYP7A1
NR1H3
CYP8B1
CYP27A1
Fatty acid transport
DBI
FABP1
SLC27A4
FABP3
CD36
LPL
OLR1
ACSL1
Fatty acid oxidation
EHHADH
CPT1A
CYP4A1
CPT2
ACAA1
ACADL
SCP2
ACADM
ACOX1
ANGPTL4
PLIN2
FABP4
ADIPOQ
SORBS1
MMP1
UCP1
ILK
PDPK1
UBC
PCK2
AQP7
GK
Fatty acid degradation
Bile acid biosynthesis
Glycerophospholipid metabolism
Lipid metabolism
Adipocyte differentiation
Adaptive thermogenesis
Cell survival
Ubiquitination
Gluconeogenesis
Ligand
Transcription factor
Target gene
Data on KEGG graph
Rendered by Pathview

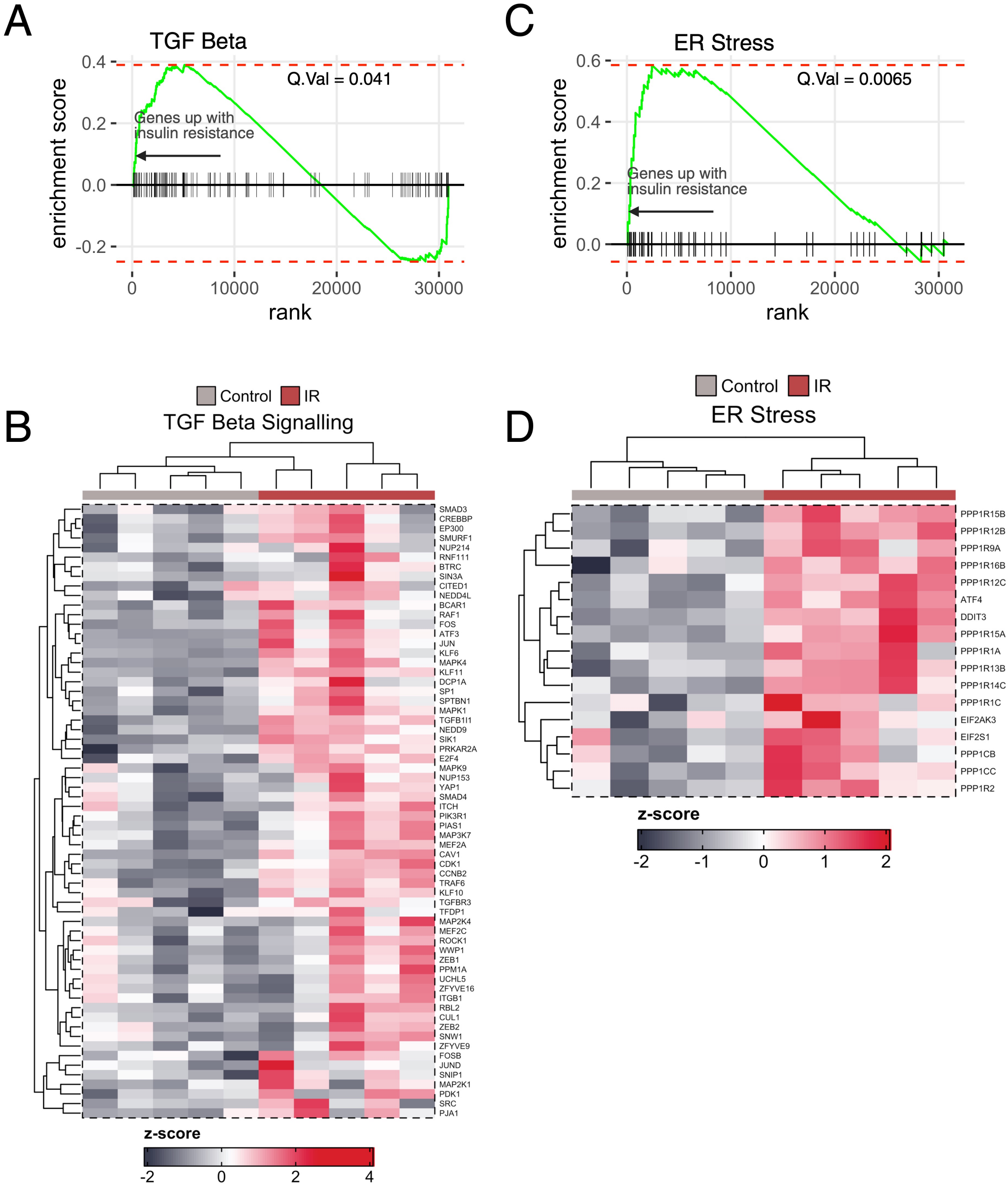
A
TGF Beta
Q.Val = 0.041
Genes up with insulin resistance
enrichment score
rank
C
ER Stress
Q.Val = 0.0065
Genes up with insulin resistance
enrichment score
rank
B
Control
IR
TGF Beta Signalling
SMAD3
CREBBP
EP300
SMURF1
NUP214
RNF111
BTRC
SIN3A
CITED1
NEDD4L
BCAR1
RAF1
FOS
ATF3
JUN
KLF6
MAPK4
KLF11
DCP1A
SP1
SPTBN1
MAPK1
TGFB1I1
NEDD9
SIK1
PRKAR2A
E2F4
MAPK9
NUP153
YAP1
SMAD4
ITCH
PIK3R1
PIAS1
MAP3K7
MEF2A
CAV1
CDK1
CCNB2
TRAF6
KLF10
TGFBR3
TFDP1
MAP2K4
MEF2C
ROCK1
WWP1
ZEB1
PPM1A
UCHL5
ZFYVE16
ITGB1
RBL2
CUL1
ZEB2
SNW1
ZFYVE9
FOSB
JUND
SNIP1
MAP2K1
PDK1
SRC
PJA1
z-score
D
Control
IR
ER Stress
PPP1R15B
PPP1R12B
PPP1R9A
PPP1R16B
PPP1R12C
ATF4
DDIT3
PPP1R15A
PPP1R1A
PPP1R13B
PPP1R14C
PPP1R1C
EIF2AK3
EIF2S1
PPP1CB
PPP1CC
PPP1R2
z-score

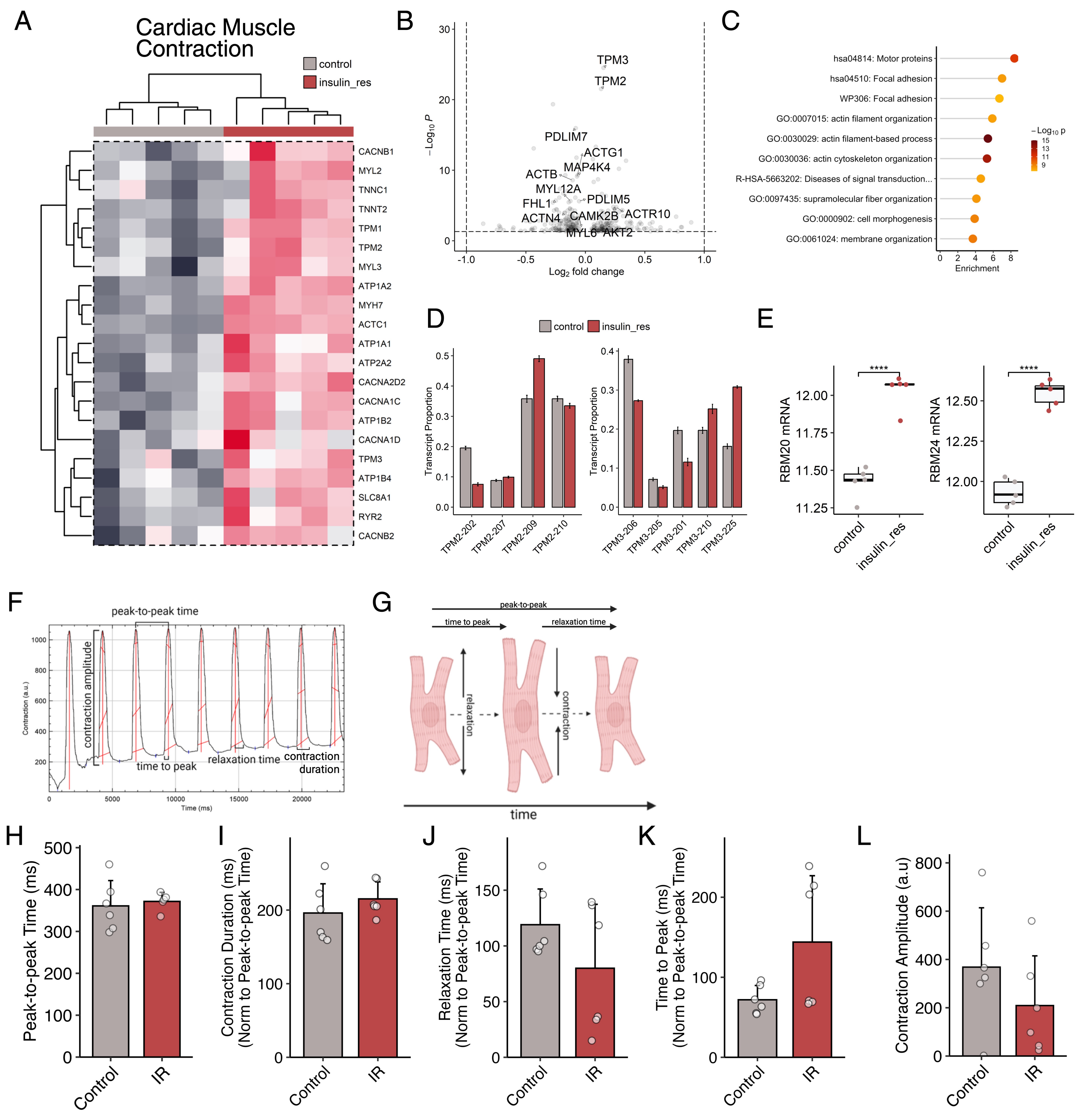
A
Cardiac Muscle Contraction
control
insulin_res
CACNB1
MYL2
TNNC1
TNNT2
TPM1
TPM2
MYL3
ATP1A2
MYH7
ACTC1
ATP1A1
ATP2A2
CACNA2D2
CACNA1C
ATP1B2
CACNA1D
TPM3
ATP1B4
SLC8A1
RYR2
CACNB2
B
TPM3
TPM2
PDLIM7
ACTG1
MAP4K4
ACTB
MYL12A
PDLIM5
FHL1
ACTR10
ACTN4
CAMK2B
MYL6
AKT2
$-\log_{10} P$
$\log_2$ fold change
C
hsa04814: Motor proteins
hsa04510: Focal adhesion
WP306: Focal adhesion
GO:0007015: actin filament organization
GO:0030029: actin filament-based process
GO:0030036: actin cytoskeleton organization
R-HSA-5663202: Diseases of signal transduction...
GO:0097435: supramolecular fiber organization
GO:0000902: cell morphogenesis
GO:0061024: membrane organization
$-\log_{10}$ p
Enrichment
D
control
insulin_res
Transcript Proportion
TPM2-202
TPM2-207
TPM2-209
TPM2-210
TPM3-206
TPM3-205
TPM3-201
TPM3-210
TPM3-225
E
RBM20 mRNA
RBM24 mRNA
****
control
insulin_res
F
peak-to-peak time
contraction amplitude
time to peak
relaxation time
contraction duration
Contraction (a.u.)
Time (ms)
G
peak-to-peak
time to peak
relaxation time
relaxation
contraction
time
H
Peak-to-peak Time (ms)
Control
IR
I
Contraction Duration (ms) (Norm to Peak-to-peak Time)
Control
IR
J
Relaxation Time (ms) (Norm to Peak-to-peak Time)
Control
IR
K
Time to Peak (ms) (Norm to Peak-to-peak Time)
Control
IR
L
Contraction Amplitude (a.u)
Control
IR

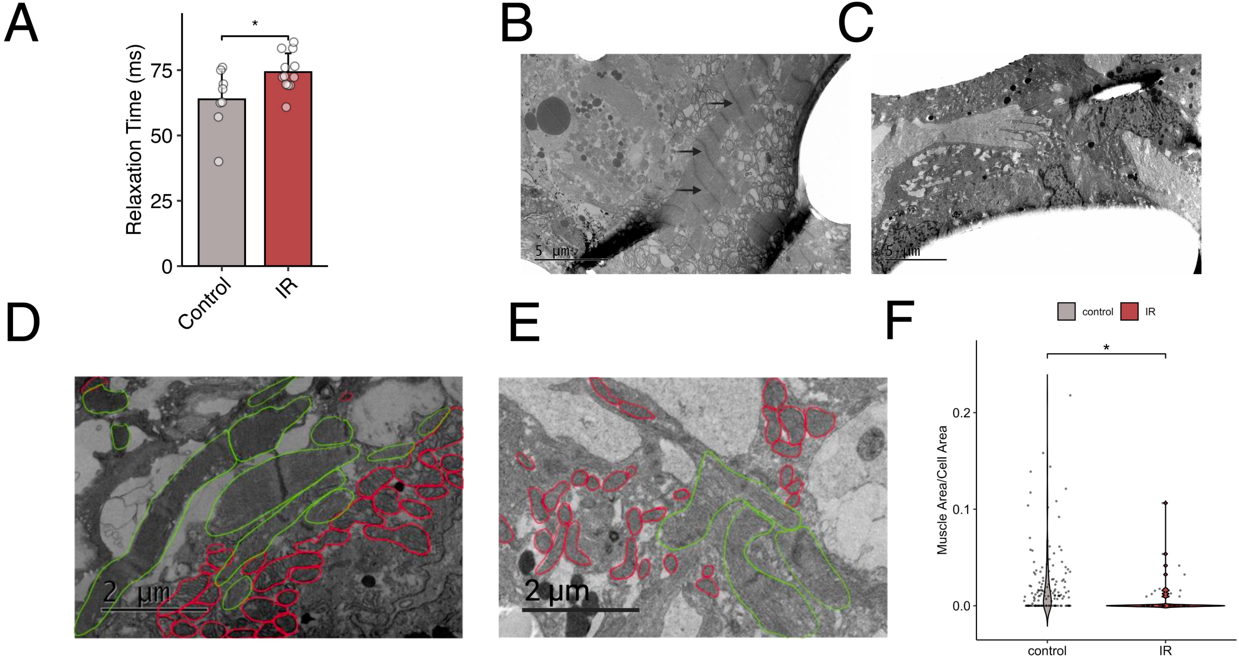

A
B
C
D
E
F
Relaxation Time (ms)
0
25
50
75
*
Control
IR
5 µm
5 µm
2 µm
2 µm
control
IR
Muscle Area/Cell Area
0.0
0.1
0.2
*
control
IR
